# The anti-senescence effect of D-β-hydroxybutyrate in Hutchinson-Gilford progeria syndrome involves progerin clearance by the activation of the AMPK-mTOR-autophagy pathway

**DOI:** 10.1007/s11357-024-01501-9

**Published:** 2025-01-16

**Authors:** Feliciano Monterrubio-Ledezma, Ashley Salcido-Gómez, Tania Zavaleta-Vásquez, Fernando Navarro-García, Bulmaro Cisneros, Lourdes Massieu

**Affiliations:** 1 Department of Neuropathology, Instituto de Fisiología Celular, Universidad Nacional Autónoma de México (UNAM), 04510 Mexico City, Mexico; 2 Department of Genetics and Molecular Biology, Centro de Investigación y de Estudios Avanzados del Instituto Politécnico Nacional (CINVESTAV-IPN), 07360 Mexico City, Mexico; 3 Department of Cell Biology, Centro de Investigación y de Estudios Avanzados del Instituto Politécnico Nacional (CINVESTAV-IPN), 07360 Mexico City, Mexico

**Keywords:** Hutchison-Gilford progeria syndrome, Ketone bodies, Senescence, Autophagy, Progerin clearance, AMPK

## Abstract

D-β-hydroxybutyrate, BHB, has been previously proposed as an anti-senescent agent in vitro and in vivo in several tissues including vascular smooth muscle. Moreover, BHB derivatives as ketone esters alleviate heart failure. Here, we provide evidence of the potential therapeutic effect of BHB on Hutchinson-Gilford progeria syndrome (HGPS), a rare condition characterized by premature aging and heart failure, caused by the presence of progerin, the aberrant protein derived from *LMNA/C* gene c.1824C > T mutation. We have assessed several hallmarks of HGPS-senescent phenotype in vitro, such as progerin levels, nuclear morphometric aberrations, nucleolar expansion, cellular senescent morphology, SA-βGal-positive cells, H3K9me3 heterochromatin, γH2AX foci, Lamin B1, p21^Waf1/Cip1^ and p16^CDKN2A^ abundance, and autophagy. Strikingly, BHB improved nuclear and nucleolar morphometrics, diminished the senescence-phenotype, and unstuck autophagy in HGPS as observed by an enhanced degradation of the cargo protein receptor SQSTM1/p62, suggesting the stimulation of the autophagic flux. Additionally, we observed a decrease in progerin abundance, the cause of senescence in HGPS. Furthermore, compound C, an inhibitor of AMPK, and SBI-0206965, an inhibitor of ULK1/2 and AMPK, which prevent autophagy activation, reversed BHB-induced progerin decline as well as its anti-senescent effect in an AMPK-mTORC1 dependent manner. Altogether, these results suggest that the anti-senescence effect of BHB involves progerin clearance by autophagy activation supporting the potential of BHB for HGPS therapeutics and further preclinical trials.

**Graphical Abstract:**

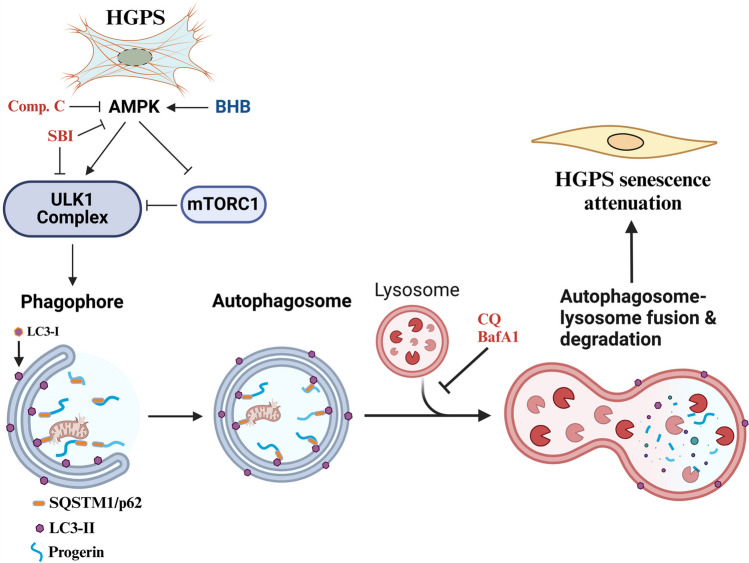

**Supplementary Information:**

The online version contains supplementary material available at 10.1007/s11357-024-01501-9.

## Introduction

Hutchinson-Gilford progeria syndrome (HGPS) is a rare condition characterized by premature aging. It is caused by a congenital mutation in exon 11 of the Lamin A/C (*LMNA)* gene (c.1824C > T), which leads to the emergence of a new splicing site. This leads to the loss of 50 amino acids, where the recognition sequence for the zinc metallopeptidase STE24 (ZMPSTE24) is encoded, producing a permanently farnesylated prelamin A, named progerin [1]. Progerin induces a plethora of cellular alterations including nuclear [2] and nucleolar abnormalities [3], epigenetic and gene expression dysregulation [4], mitochondrial and lysosomal dysfunction [5], endoplasmic reticulum stress (ER stress) [6], and premature cell senescence [7]. Senescence is a hallmark of aging, characterized by permanent cell cycle arrest, and the acquisition of a senescence-associated secretory phenotype (SASP). The most used senescence markers include p21 and p16 abundance, SA-βGal activity, Lamin B1 decreased content, and heterochromatin loss [8].

HGPS patients start to show an aging phenotype at two years old, and their average life expectancy is 14.5 years. The common cause of demise is heart failure or stroke [1, 9]. Nowadays, there is only one treatment for HGPS, which is based on lonafarnib, a farnesyl transferase inhibitor (FTI) that prevents prelamin-A farnesylation, interrupting progerin synthesis. Although lonafarnib has some beneficial effects on HGPS patients such as cardiovascular improvement, lifespan, and health span extension, these effects are limited. Lonafarnib only extends lifespan by 1.6 years and health span by 2.5 to 2.8 years [10, 11]. Moreover, FTI treatment seems suitable for progerin synthesis inhibition but not for its clearance, limiting its therapeutic effects to early stages of the syndrome [12]. Therefore, the search for new and more effective treatments is ongoing.

D-β-hydroxybutyrate (BHB) is a ketone body derived from acetyl-CoA produced in hepatic mitochondria by a process called ketogenesis under low blood glucose conditions, such as prolonged fasting, strenuous exercise, ketogenic diet, caloric restriction, and glucose transporter blockade [13, 14]. The ketone bodies, BHB and acetoacetate, are released from the liver to the bloodstream and transported to peripheral tissues through monocarboxylate transporters, where they are converted to Acetyl-CoA to produce energy. Beyond its metabolic role, BHB regulates several cellular processes potentially involved in the establishment of senescence. For example, proteins such as histones [15] or p53 [16] can be β-hydroxybutyrylated, changing gene expression or reducing p21 abundance [16]. Additionally, BHB acts on heterogeneous nuclear ribonucleoprotein A1, thereby alleviating vascular and cartilage senescence via Oct4 [17] and PTEN [18], respectively. BHB can also inhibit histone deacetylase activity and modulate the antioxidant response [19]. Furthermore, studies have reported the extension of lifespan after the ketogenic diet in mice and the administration of the BHB salt in *C. elegans* [20, 21].

On the other hand, BHB regulates autophagy [22–28], a degradative process that turns damaged proteins and cellular organelles into simpler molecules for cellular utilization [29]. Commonly used markers of autophagy include the conversion of LC3-I (microtubule-associated protein 1A/1B-light chain 3) to LC3-II, a protein involved in autophagosome formation, and the degradation of the cargo receptor, sequestosome 1 (SQSTM1/p62). Thus, an increase in LC3-II and a reduction in SQSTM1/p62 indicates an efficient autophagosome formation and cargo degradation, also referred to as autophagic flux [30]. In HGPS, autophagy is impaired [31–33], as well as other selective autophagy processes like mitophagy [5, 34]; thus, strategies aimed towards autophagy activation commonly lead to the alleviation of HGPS senescence phenotype, in most cases by the induction of progerin clearance [31, 35–37]. For example, autophagy activation by rapamycin (the mammalian target of mTOR) [37]; MG132, a proteasome inhibitor [38]; progerinin [39, 40]; 991, an inducer of AMPK [31]; A485, an inhibitor of the acetyltransferase, p300 [31]; neuropeptide Y [41]; sulforaphane [32]; and ghrelin [33] drive progerin clearance and further alleviation of the progerin-induced senescent phenotype. In fact, a previous study showed that BHB reduces the senescence phenotype through stimulation of the autophagic flux in the liver of male rats [42].

The main regulator of autophagy is the mammalian target of rapamycin (mTOR), whose inactivation leads to autophagy induction; this commonly occurs via 5′AMP-activated protein kinase (AMPK) in conditions of nutrient depletion (low amino acids and glucose and high AMP/ATP ratio) [43]. Also, AMPK stimulates autophagy initiation by the direct phosphorylation of Unc-51-like kinase 1 (ULK1) at the serine 317 residue [43]. Moreover, BHB has been found to activate AMPK in mice’s liver and in the central nervous system (CNS) [23, 44]. In the latter, activation of AMPK by BHB is involved in autophagy stimulation.

In the present study, we aimed to test whether BHB can alleviate HGPS senescence and if this effect correlates with autophagy induction. With this purpose, we treated HGPS-derived fibroblasts with BHB and measured the abundance of progerin, as well as markers of the senescence phenotype and autophagy. We report that BHB treatment reduces the abundance of progerin and several progerin-induced alterations, like nuclear envelope aberrations, nucleoli expansion, and other senescence markers. Moreover, we show that autophagy is also improved by BHB. Progerin decrease, autophagy flux improvement, and the anti-senescence effect observed after BHB administration are reversed by AMPK-ULK1 inhibition suggesting an AMPK-ULK1-mediated mechanism; moreover, these effects are comparable to those of rapamycin, an autophagy inducer through mTOR inhibition.

## Methods

### Cell culturing and drug treatment

Human primary dermal fibroblast, from 23-, 38-, and 34-year-old healthy donors, GM04390 (CTL-23y), AG08469 (CTL-38y), and AG06299 (CTL-34y), respectively, and HGPS-affected primary dermal fibroblast carrying the classic G608G splice site mutation at the LMNA/C gene: AG06917 (HGPS-3y), AG11513 (HGPS-8y), AG11498 (HGPS-14ya), and AG01972 (HGPS-14yb) from 3, 8, and 14 years old (age at the time of sample), respectively, were acquired from Coriell Cell Repositories (Camden NJ). Fibroblasts were cultured in a humidified 5% CO_2_ atmosphere in Minimal Essential Medium Eagle (MEM) (Invitrogen, Carlsbad, CA, USA) supplemented with 10% fetal bovine serum (Invitrogen, USA), pyruvate 1 mM (Sigma, Irvine, UK), 10 U/mL penicillin and 10 µg/mL streptomycin (Sigma, Saint Louis, MO, USA). When indicated, fibroblast cultures were treated with 3 mM BHB sodium salt (BHB; Merck Millipore, MO, USA) and 50 μM chloroquine (CQ; Sigma, Saint Louis, MO, USA) solubilized in water, 1.5 µM doxorubicin hydrochloride (Sigma, Saint Louis, MO, USA [D1515]), 200 nM bafilomycin A1 (Caiman chemical, MI, USA, 5 μM compound C, 10 μM SBI-0206965 (SBI; Merk, Darmstadt, Germany [SML1540]), 1 μM rapamycin (Thermo-Fisher Scientific, USA [PHZ1235]), and 0.5 μM MG132 (Sigma, Saint Louis, MO, USA [474790]) solubilized in DMSO. Fresh medium was added every 2 days, and cultures were passaged at 95% confluency at a 1:3 ratio by trypsinization (0.05% of trypsin–EDTA, Invitrogen, Carlsbad, CA, USA [15400054]). Fibroblast cell cultures were treated with BHB for 24 or 72 h, and media with treatment was changed every 2 days. When indicated, fibroblasts were treated with BHB alone or along with BafA1, compound C, or SBI for 24 h. CQ was added during the last 12 h of BHB treatment. Cells were exposed to doxorubicin hydrochloride for 1 h, and then fresh cell cultured media was added for 2 h prior to fixation. Rapamycin or MG132 was added for 72 h.

### Antibodies

The following primary antibodies were used: mouse monoclonal anti-progerin antibody, 1:1000 for WB and 1:50 for IF (Santa Cruz Biotechnology, CA, USA [SC-81611]); mouse monoclonal anti-Lamin A/C antibody, 1:2000 for WB and 1:200 for IF (Hybridoma Bank [MANLAC3-4C10]); rabbit polyclonal anti-H3K9me3 antibody, 1:50 for IF (Abcam, LA, USA [Cat: ab8898]); rabbit polyclonal anti-B23 antibody, 1:150 for IF (Santa Cruz Biotechnology, CA, USA [sc − 6013‐a]); rabbit polyclonal anti-Lamin B1 antibody, 1:1000 for WB and 1:200 for IF (Abcam, LA, USA [ab16048]); mouse monoclonal anti-p21^Waf1/Cip1^, 1:1000 for WB (Cell Signaling Technology, MA, USA [2946S]); mouse monoclonal anti-p16^CDKN2A^ antibody, 1:1000 for WB (Santa Cruz Biotechnology, CA, USA [sc − 1661]); rabbit polyclonal anti-LC3 antibody, 1:2000 for WB and 1:200 for IF (MBL International, Woburn, MA, USA [cat: PD014]); mouse monoclonal anti-actin antibody, 1:10,000 for WB (Merck Millipore, MO, USA [MAB1501]); mouse monoclonal anti-SQSTM1/p62, 1:3,000 (Abcam, LA, USA [ab56416]); rabbit polyclonal anti-p-ULK1 (Ser317), 1:1,000 (MyBioSourse, SA, USA [MBS9600629]; rabbit polyclonal anti-ULK1, 1:1,000 (Cell Signaling Technology, MA, USA [8054]); rabbit polyclonal anti-p-S6K1 (Thr389), 1:1000 (Cell Signaling Technology, MA, USA [9205]); rabbit polyclonal anti-S6K1, 1:2,000 (Cell Signaling Technology, MA, USA [2708]); mouse monoclonal anti-γH2AX antibody, 1:250 for IF (Merck Millipore, MO, USA [05–636]).

### Western blotting (WB)

Fibroblast protein lysates were electrophoresed in SDS–polyacrylamide gels and transferred onto polyvinylidene fluoride (PVDF) membranes (Immobilon-P Membrane, Merck Millipore, MO, USA [IPVH00010]). The membranes were blocked in TBST [100 mM Tris–HCl pH 8.0, 150 mM NaCl, 0.5% (v/v) Tween-20] with 5% low-fat milk and incubated overnight at 4 °C with the primary antibody. The specific protein signal was developed using the correct secondary antibodies and the enhanced chemiluminescence (ECL™) Western blotting (WB) detection system (C-DiGit Scanner (LI-COR). The ImageJ software version 2.1.0 (Wayne Rasband (NIH), USA) was used to analyze images.

### Immunofluorescence (IF) and confocal microscopy

Cells were grown on coverslips at 70% confluence, and then they were washed with PBS and incubated for 20 min with 4% paraformaldehyde at room temperature. Coverslips were incubated with 5% albumin-PBS and 0.1% Triton X-100 for 1 h at room temperature. Afterwards, cells were incubated overnight at 4 °C with the appropriate primary antibodies diluted in 5% albumin-PBS and 0.1% Triton X-100 and then with the corresponding secondary fluorochrome-conjugated antibodies for 1 h at room temperature (Jackson Immunoresearch Laboratories, West Grove, PA, USA). Nuclei were stained with 0.001% Hoechst (Sigma-Aldrich, 33,258) or 1 mg/mL diamidino-2-phenylindole (DAPI) (Sigma-Aldrich, St. Louis, MO, USA), both in PBS, when it is indicated. For cellular morphology experiments, we used rhodamine phalloidin reagent (Abcam, LA, USA [ab235138]) as follows: fibroblasts were seeded in coverslips; they were treated or not with BHB, then washed with PBS and fixed with PFA 4% for 10 min, washed again with PBS, and incubated with 5% albumin-PBS and 0.1% Triton X-100 for 15 min at room temperature. Cells were stained with rhodamine phalloidin at 1:150 for 15 min at room temperature, and nuclei were stained with DAPI. Finally, the coverslips were mounted with Fluoromount-G™ (Electron Microscopy Sciences, 17984–25, Hatfield, PA, USA) for image acquisition using the inverted Zeiss LSM 800 confocal microscope and analyzed using ImageJ. For morphometric nuclear analysis, we used the ImageJ plug-in nuclear irregularity index (NII) to obtain the radius ratio and nuclear contour values [45]. The radius ratio is defined as the ratio between the maximum and the minimum radius of the object and the nuclear contour as the roundness (border) calculated by the perimeter^2^/(4 ∗ pi ∗ area) [45].

### Senescence‐associated β‐galactosidase (SA β‐Gal) assay

Fibroblasts seeded on p35 dishes were washed with PBS and stained with X-Gal stain solution (X-Gal 1 mg/ml; sodium phosphate/citric acid buffer 40 mM; K_4_[Fe(CN)_6_] 5 mM; K_4_[Fe(CN)_6_]·3 H_2_O 5 mM; NaCl 150 mM; MgCl 2 mM) at 37 °C for 12 h. Then, cells were washed with PBS, and nuclei were stained with 0.001% Hoechst/PBS for total cell counting. Blue‐stained-positive cells for senescence-associated β‐galactosidase activity (senescent cells) were observed and counted using an epifluorescence microscope (Olympus IX71). Finally, the percentage of SA β-Gal-positive cells/total cells was calculated for each group.

### Cell proliferation assay

Fibroblasts seeded on 12-well dishes were treated or not with BHB 3 mM and harvested by trypsinization at 0, 48, and 96 h for viable cell counting using a Neubauer chamber.

### Live/dead assay

The LIVE/DEAD Viability/Cytotoxicity Kit for mammalian cells (Thermo-Fisher Scientific, USA [L3224]) was used to assess cell viability. Fibroblasts seeded on 12-well dishes were treated or not with BHB 3 mM for 72 h, then 1 µl per ml of calcein AM (50 μM) and 1 µl per ml of ethidium homodimer-1 (EthD-1; 2 mM) were added, and nuclei were stained with 0.001% Hoechst and incubated by 30 min. The cell culture media was removed, and CO_2_-saturated Lockey buffer was added for epifluorescence microscopy observation (Nikon Eclipse Ti). EthD-1-positive cells were considered dead cells, and calcein-positive cells were considered living cells. In total, 0.3% Triton was employed as a control of cell death as membrane permeabilization allows EthD-1 influx and nuclei staining.

### Statistical analysis

The data are expressed and analyzed as described in each caption. The student test was used for means comparisons and the Mann–Whitney test for rank comparisons. An unpaired two-way ANOVA test was used to compare the percent nucleoli per nucleus. Statistical significance of *p* ≤ 0.05 and an interval confidence of 95% were used. All statistical analyses and graphs were performed using GraphPad Prism 8 software.

## Results

### BHB treatment reduces progerin protein levels in human HGPS primary fibroblasts

**Fig. 1:**
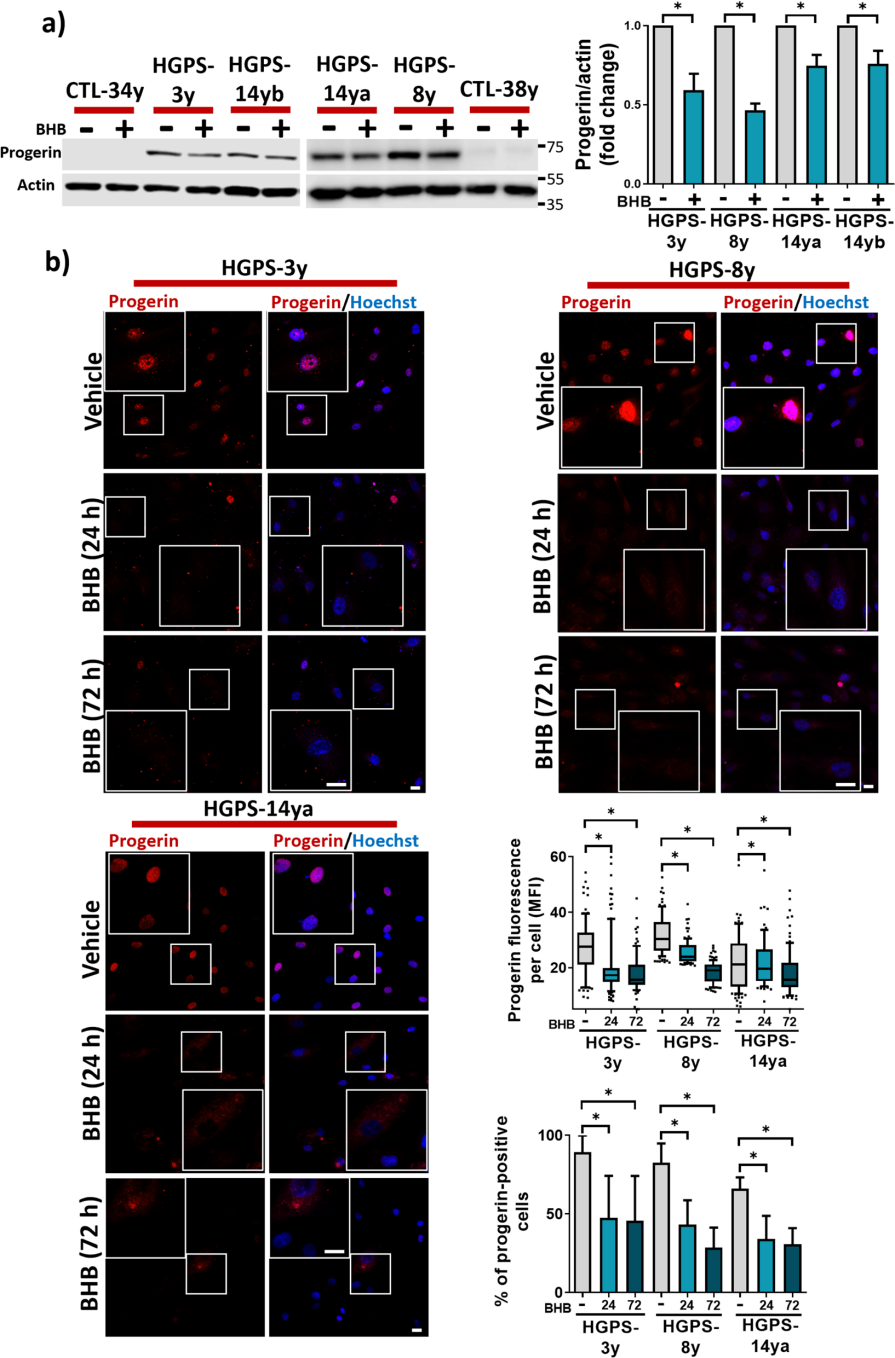
BHB treatment diminished progerin protein abundance in HGPS primary fibroblast. **a**: *Left panel*, WB showing progerin abundance in fibroblast cultures from HGPS patients and healthy individuals treated or not with 3 mM BHB using anti-progerin and anti-actin (loading control) antibodies; *right panel*, the graph shows the relative protein levels of progerin as mean ± SD from three independent experiments. Statistical differences were calculated using the unpaired t-test; *p < 0.05. The mean value obtained in each vehicle-treated HGPS cell culture was set at 1, and the mean value of BHB-treated HGPS cells was normalized relative to the vehicle. **b** *Left panel*, progerin IF in HGPS cell cultures treated with 3 mM BHB or vehicle for 24 and 72 h. Nuclei were stained with Hoechst; scale bar 20 µm. *Bottom right panel*, the mean progerin immunofluorescence intensity (MFI) per cell was quantified, and data from three independent experiments are shown in a box and whiskers graph (percentile 10–90, *n* = 100 cells per condition), and the percent of progerin-positive cells (mean ± SD, *n* = 100 cells per condition) is shown in the bar graph below. Statistical differences were calculated using the nonparametric Mann–Whitney test and the unpaired *t*-test, respectively; *p < 0.05

Since progerin abundance is a determinant for HGPS-senescent phenotype, we first analyzed whether BHB treatment changes progerin levels. Using a specific anti-progerin antibody, we found by immunoblot that progerin abundance diminished after 3 days of BHB treatment in all HGPS fibroblast cultures (Fig. 1a). Consistently, we were able to detect a significant decline in progerin content after BHB treatment using an anti-Lamin A/C antibody in cultures from HGPS-8y; we also observed a Lamin A decline in CTL-38y upon BHB treatment (Fig S1a). Moreover, progerin content as well as the percentage of progerin-positive cells diminished as soon as 24 h after BHB treatment and persisted at 72 h in HGPS-3y, HGPS-8y, HGPS-14ya, and HGPS-14yb as measured by IF (Fig. 1b and Fig S1b). These results suggest that BHB incubation after 24 and 72 h can reduce the levels of progerin.

### BHB treatment reduces nuclear and nucleolar abnormalities in HGPS primary fibroblast cultures

**Fig. 2:**
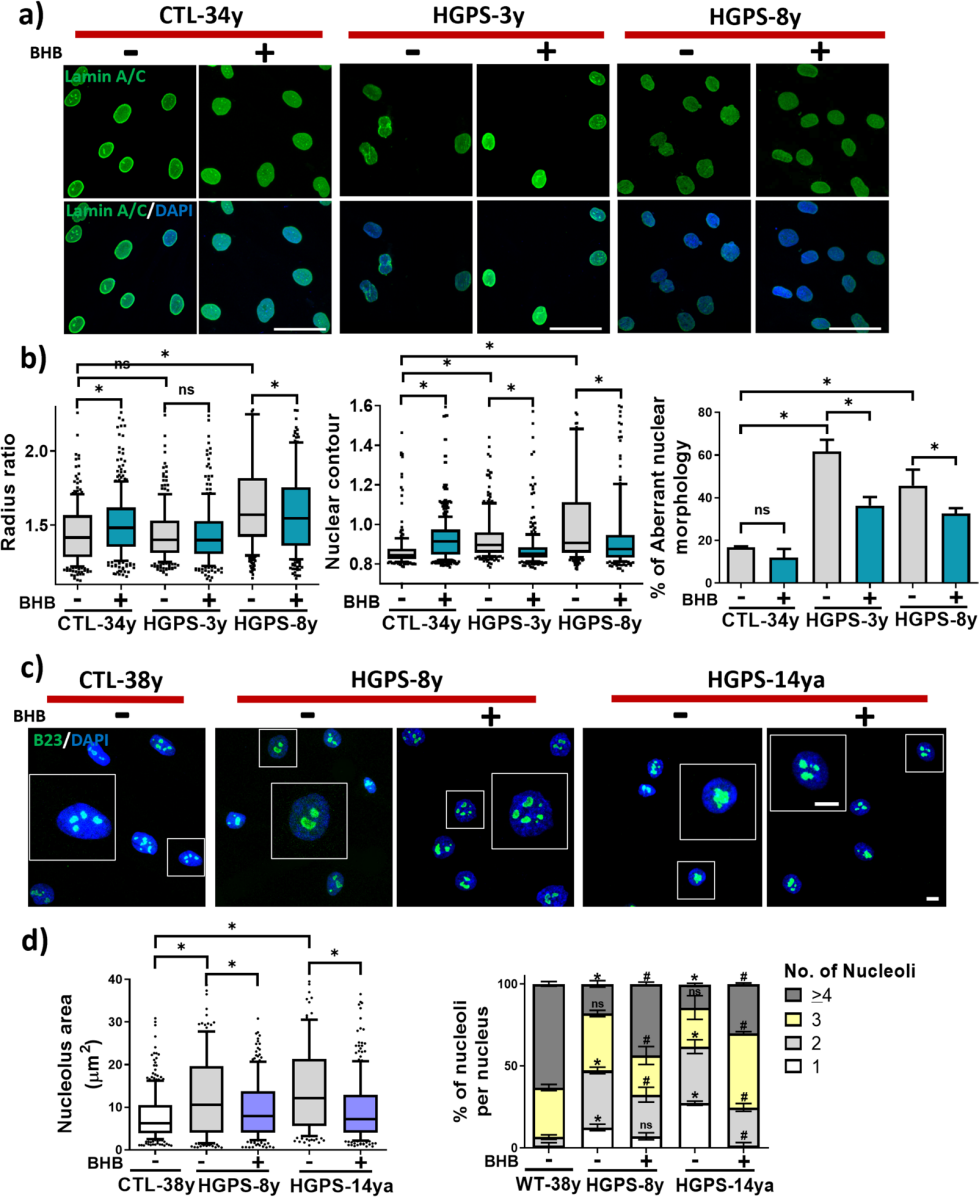
BHB treatment diminished nuclear envelope abnormalities and nucleolar expansion. **a** IF of Lamin A/C in CTL-34y and HGPS-3y and HGPS-8y cell cultures treated or not with 3 mM of BHB for 72 h. Nuclei were stained with DAPI; scale bar 25 µm. **b** *Left*, radius ratio, and *middle*, nuclear contour data per cell quantified using NII plug-in from three independent experiments per group. Data were plotted in a box and whiskers graph (percentile 10–90, *n* = 300 cells per condition). Significant differences were obtained using the nonparametric Mann–Whitney test; *p < 0.05; ns, no statistical significance. *Right*, qualitative analysis of nuclear aberrant morphology. The percentage of aberrant nucleus from three independent experiments per group was plotted and shown as the mean ± SD (*n* = 300 cells per condition). Statistical differences were calculated using the unpaired *t*-test test; *p < 0.05. **c** IF of nucleophosmin/B23 in CTL-38y, HGPS-8y, and HGPS-14ya cell cultures, treated or not with 3 mM BHB for 72 h. Nuclei were stained with Hoechst; scale bar 10 µm. **d** *left panel*, the nucleolus area from three independent experiments per group, plotted in a box and whiskers graph (percentile 10–90, *n* ≈ 100 cells per condition). Statistical differences were calculated using the nonparametric Mann–Whitney test; *p < 0.05. *Right panel*, the number of nucleoli per cell from three independent experiments per group, plotted in a stacked column graph and shown as the percentage mean ± SD (*n* = 90 cells per condition). Statistical differences were calculated using the unpaired two-way ANOVA test; healthy-38y non-treated vs HGPS-8y and HGPS-14ya non-treated, *p < 0.05; HGPS-8y non-treated vs HGPS-8y BHB-treated, and HGPS-14ya non-treated vs HGPS-14ya BHB-treated. #*p* < 0.05; ns, no significant

The HGPS most prominent cellular signature is an abnormal nuclear morphology; therefore, we assessed nuclear morphometrics after BHB treatment in HGPS-3y, HGPS-8y, HGPS-14ya, and healthy individuals, CTL-34y cell cultures, using anti-Lamin A/C antibody for nuclear envelope labeling by IF, to test if BHB could reduce progerin-associated nuclear abnormalities. With this purpose, we performed a nuclear morphometric analysis by measuring the radius ratio and the nuclear contour [45, 46], as well as the proportion of abnormal nuclei containing slits, bumps, blebbing, and corrugations. As shown in Fig. 2a, b, the radius ratio of HGPS-8 was higher with respect to CTL-34y nuclei, and it diminished after BHB treatment. In HGPS-3y fibroblast, the radius ratio was not statistically different from that observed in CLT-34y cells (Fig. 2b). Regarding the nuclear contour, it is elevated in HGPS-3y and HGPS-8y with respect to CTL-34y fibroblasts and decreases after BHB treatment (Fig. 2a, b). Moreover, qualitative analysis shows an elevation of the percentage of abnormal nuclei in both HGPS cell cultures, with respect to cultures from healthy individuals, and this percentage diminishes in HGPS-3y and HGPS-8y after BHB treatment (Fig. 2a, b).

Due to the nucleolar area expansion observed in HGPS senescent phenotype [3], we also performed a nucleolar analysis after BHB treatment, to assess the nucleolar area and fragmentation. To this end, we employed an antibody directed to nucleophosmin/B23 protein, a classic marker of the nucleoli granular center [47]. Then, using confocal images, the area from each nucleolus and the nucleoli number per cell (divided in four categories 1, 2, 3, and ≥ 4 nucleoli per cell) were quantified. The results show that in HGPS-8y and HGPS-14ya, the nucleolar area augmented compared to CTL-38y, along with a reduction in the percent of nuclei containing ≥ 4 nucleoli and the elevation of the percentage of nuclei containing 1 and 2 nucleoli (Fig. 2c, d), consistently with previous reports [3, 46]. After BHB treatment, the nucleolar area diminished along with an elevation in the percentage of nuclei containing ≥ 4 nucleoli in HGPS-8y and HGPS-14ya cell cultures and an elevation of nuclei with the 3 nucleoli in HGPS-14ya-treated group (Fig. 2d). Also, we observed a reduction in the percent of nuclei containing 2 and 3 nucleoli in the HGPS-8y BHB-treated group and a reduction in nuclei with 1 and 2 nucleoli in the HGPS-14ya BHB-treated group, when compared to non-treated HGPS controls, respectively (Fig. 2d). Then, we wondered whether the nucleolus area changes in HGPS treated or untreated with BHB. With this purpose, we took the data from the nucleolar area analysis and clustered it in five categories according to nucleoli size: < 10 μm^2^, 10 < 20 μm^2^, 20 < 30 μm^2^, 30 < 40 μm^2^, 40 μm^2^ < . We observed a lower percent of < 10 μm^2^ nucleoli and a higher percent of 30 < 40 μm^2^ and ≤ 40 μm^2^ categories in HGPS-8y and HGPS-14ya compared with CTL-38y. After BHB treatment, this was partially reversed (Fig. S2a). So far, these data indicate that nuclear and nucleolar morphology and morphometric parameters are restored after BHB treatment in HGPS fibroblasts.

### BHB treatment reduces the senescent phenotype in HGPS fibroblast

**Fig. 3:**
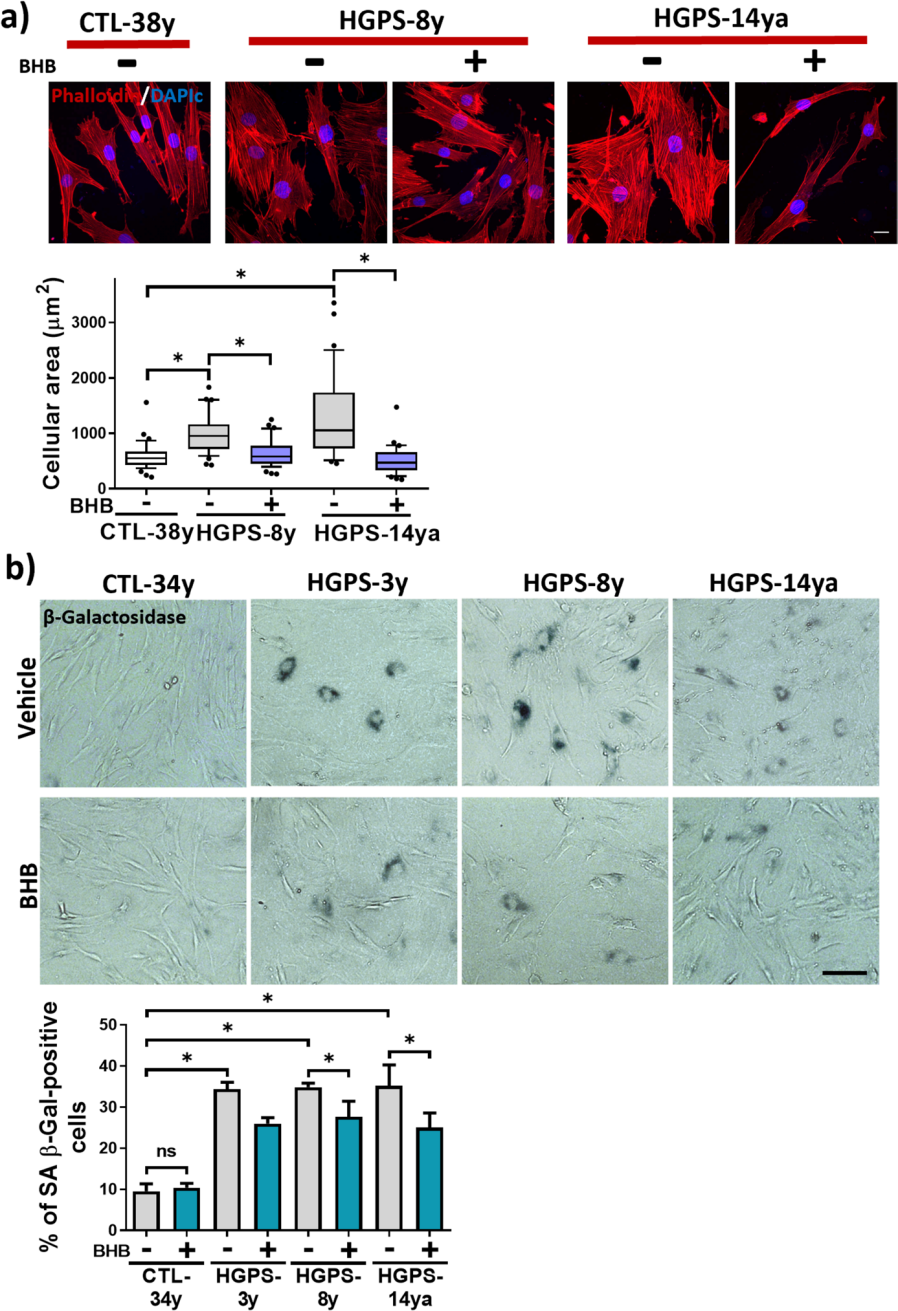
BHB reduces senescence and cellular senescent morphology.** a** *Upper panel*, representative images of rhodamine phalloidin IF from CTL-38y, HGPS-8y, and HGPS-14ya fibroblast cultures treated or not with 3 mM BHB for 72 h. Nuclei were stained with DAPI; scale bar 10 µm. *Lower panel*, graph shows the cellular area obtained using ImageJ from three independent experiments per group plotted in a box and whiskers graph (percentile 10–90, 60 cells per condition). Statistical differences were calculated using the nonparametric Mann–Whitney test; *p < 0.05. **b** *Upper panel*, representative images of SA β-Gal-positive cells (blue) in CTL-34y, CTL-38y, HGPS-3y, HGPS-8y, and HGPS-14ya fibroblast cultures treated or not with 3 mM BHB for 72 h (scale bar = 60 μm). *Lower panel*, the percentage of SA β-Gal-positive cells plotted as mean ± SD (*n* = 300 cells per condition from three independent replicates per group). Statistical differences were calculated using the unpaired *t*-test test; *p < 0.05; ns, no statistical significance

The HGPS therapeutical strategies, such as treatment with progerinin [39], antisense morpholino [48], rapamycin [37], and MG132 [38] lead to the alleviation of senescence in HGPS by diminishing progerin. Due to progerin reduction after BHB exposure, we aimed to evaluate the senescent status in HGPS fibroblast after BHB treatment. With this purpose, we assessed the following senescence markers: SA β-Gal staining, Lamin B1, p21^Waf1/Cip1^ and p16^CDKN2A^ protein abundance, the cellular area expansion, and heterochromatin loss. First, we sought to test whether the classical in vitro senescent cellular morphology also changes with BHB treatment [49]. As reported [46], HGPS-8y and HGPS-14ya cells exhibited an increased cellular area, and this was reversed by BHB treatment (Fig. 3a). Through the SA β-Gal assay, we found a notorious elevation of senescent cells in HGPS-3y, HGPS-8y, and HGPS-14ya fibroblasts when compared to CTL cells, but after BHB treatment, the percentage of senescent cells in HGPS-3y, HGPS-8y, and HGPS-14ya significantly decreased (Fig. 3b).

**Fig. 4:**
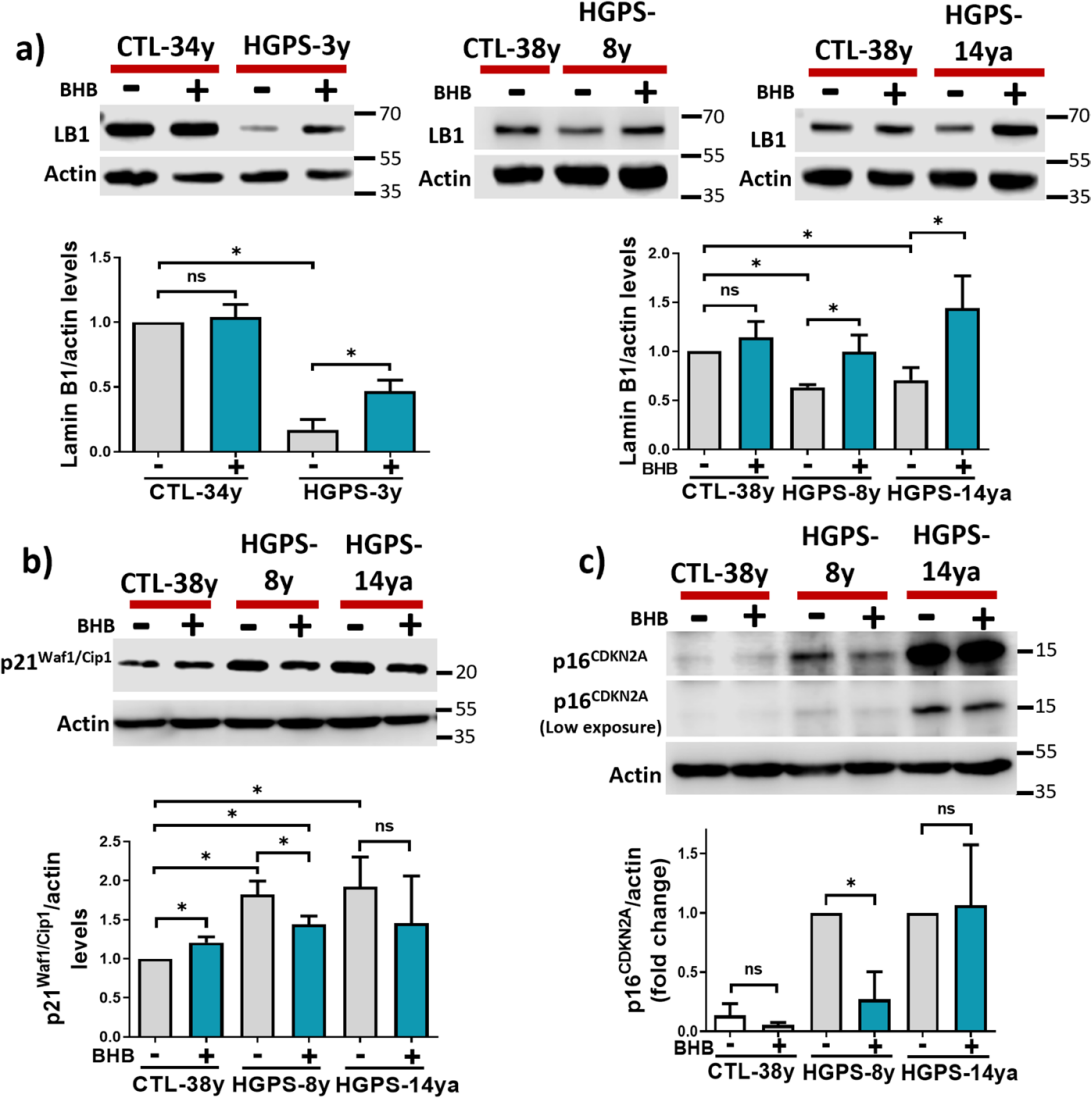
BHB restores Lamin B1 and reduces p21^Waf1/Cip1^ and p16^CDKN2A^ in HGPS.** a** *Upper panel*, representative WB showing Lamin B1 protein abundance in CTL-34y, CTL-38y, HGPS-3y, HGPS-8y, and HGPS-14ya fibroblast cultures treated or not with 3 mM BHB for 72 h. Actin was used as a loading control. *Graphs show* Lamin B1 normalized abundance expressed as mean ± SD (three independent replicates per group). The mean value obtained in vehicle-treated CTL cells was set as 1, and the mean value of the treated HGPS was normalized relative to this value. **b** and **c** *Upper panel*, representative WB showing p21^Waf1/Cip1^ and p16^CDKN2A^ protein abundance in CTL-38y, HGPS-8y, and HGPS-14ya fibroblast cell cultures treated or not with 3 mM BHB for 72 h. Actin was used as a loading control. Graphs show p21^Waf1/Cip1^ and p16^CDKN2A^ abundances plotted as mean ± SD (three independent replicates per group). For p21 ^Waf1/Cip1^ and p16^CDKN2A^, the mean value obtained in vehicle-treated CTL cells was set as 1, and the mean value of the treated HGPS was normalized to this value. Statistical differences were calculated using the unpaired *t*-test test; *p < 0.05; ns, no statistical significance

Likewise, Lamin B1 content was decreased in HGPS-3y, HGPS-8y, and HGPS-14ya fibroblasts when compared to healthy cells as has been reported in senescent cells [50], but after BHB treatment, the Lamin B1 protein levels significantly increased in all HGPS cell cultures (Fig. 4a). Then, we assessed the senescence markers p21^Waf1/Cip1^ and p16^CDKN2A^ [51], as expected both proteins showed elevated levels in HGPS-8y and HGPS-14ya cell cultures when compared to CTL cells. Upon BHB treatment, p21^Waf1/Cip1^ and p16^CDKN2A^ significantly diminished in the HGPS-8y cell culture (Fig. 4b, c).

**Fig. 5:**
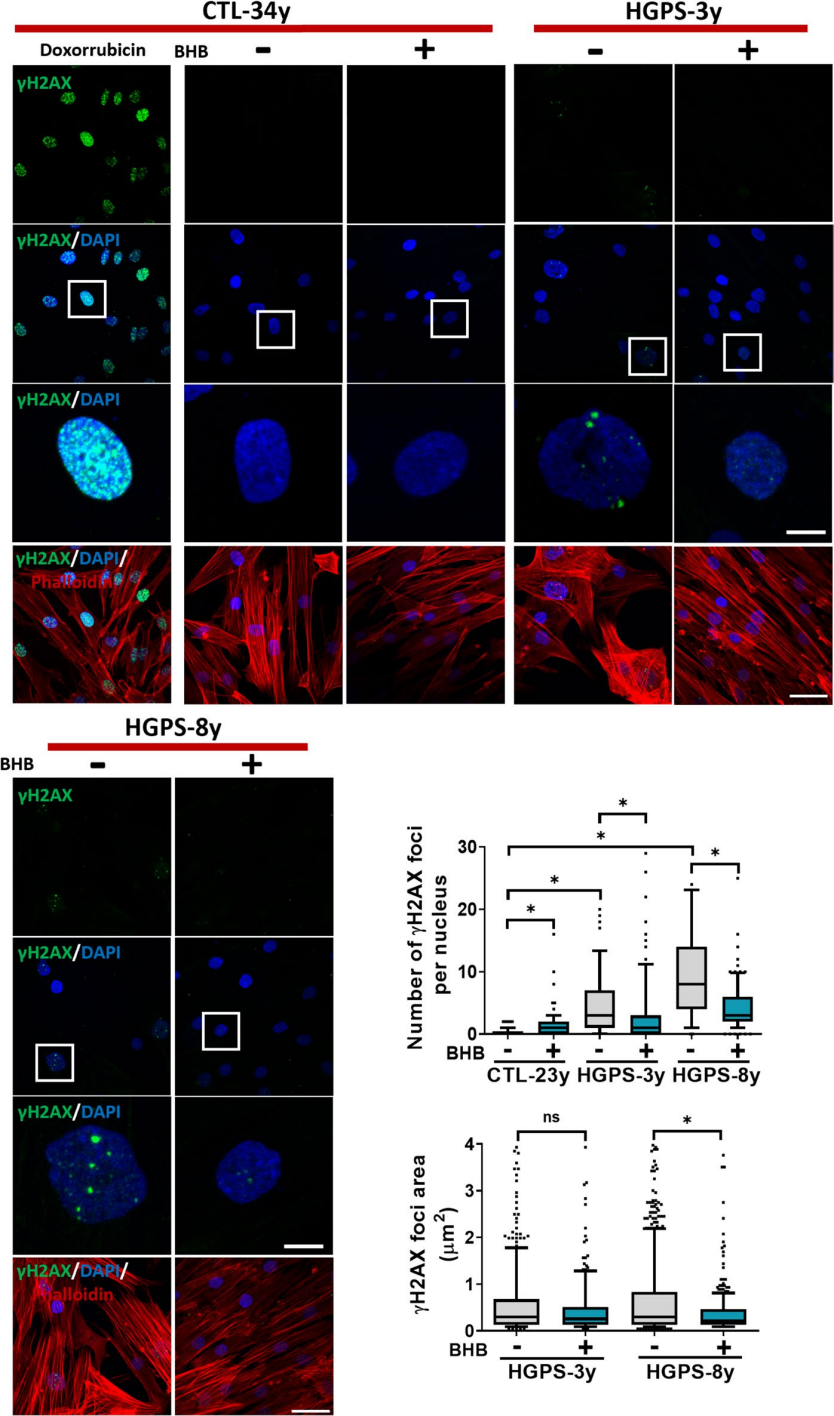
BHB reduces DNA damage. *Upper panel*, representative IF images using anti-γH2AX antibody and rhodamine phalloidin from CTL-23y, HGPS-3y, and HGPS-8y fibroblast cultures treated or not with 3 mM BHB by 72 h. Nuclei were stained with DAPI; scale bar 50 µm (panels 1, 2 and 4), zoom scale bar 10 µm (panel 3). *Lower panel*, graphs show the γH2AX foci area and the number of γH2AX foci per nucleus. The data was obtained using ImageJ and plotted in a box and whisker graph (percentile 10–90, 60 cells per condition). Statistical differences were calculated using the nonparametric Mann–Whitney test; *p < 0.05

Additionally, we analyzed the effect of BHB on heterochromatin mark H3K9me3, which is decreased in senescent cells [52], and the changes in DNA damage response, which is altered in HGPS leading to γH2AX foci accumulation [53, 54]. Consistently, in HGPS-3y and HGPS-14ya cell cultures, the H3K9me3 mark was diminished compared to CTL, and an increase in this mark was observed only in HGPS-3y cell cultures after BHB treatment (Fig. S2b). Regarding DNA damage, the γH2AX foci were evaluated by IF. It was found that the number of γH2AX foci decreased in HGPS-3y and HGPS-8y after BHB treatment, as well as the foci area in HGPS-8y (Fig. 5). Cell viability was also evaluated in CTL-34y and HGPS-8y fibroblasts by the live/dead assay, and cell proliferation was assessed by cell counting in cell cultures treated or not with BHB. It was found that the percentage of dead cells remains unaltered when HGPS cultures are compared with CTL cells. BHB had no effect on cell viability in HGPS cells; however, cell death slightly increased in CTL cells after BHB treatment (Fig. S3a). Regarding cell proliferation, a decrease in HGPS cells was observed when compared to CTL cells, but after BHB treatment, proliferation increases in HGPS and slightly decreases in CTL cells (Fig. S3b). Altogether, these data demonstrate that BHB alleviates senescent-HGPS phenotype, maintains cell viability, and restores cell proliferation, specifically in the HGPS-3y and HGPS-8y cell cultures, which correlates with the reduction of progerin levels.

### BHB-induced decrease in progerin levels in HGPS primary fibroblast is mediated by autophagy

**Fig. 6:**
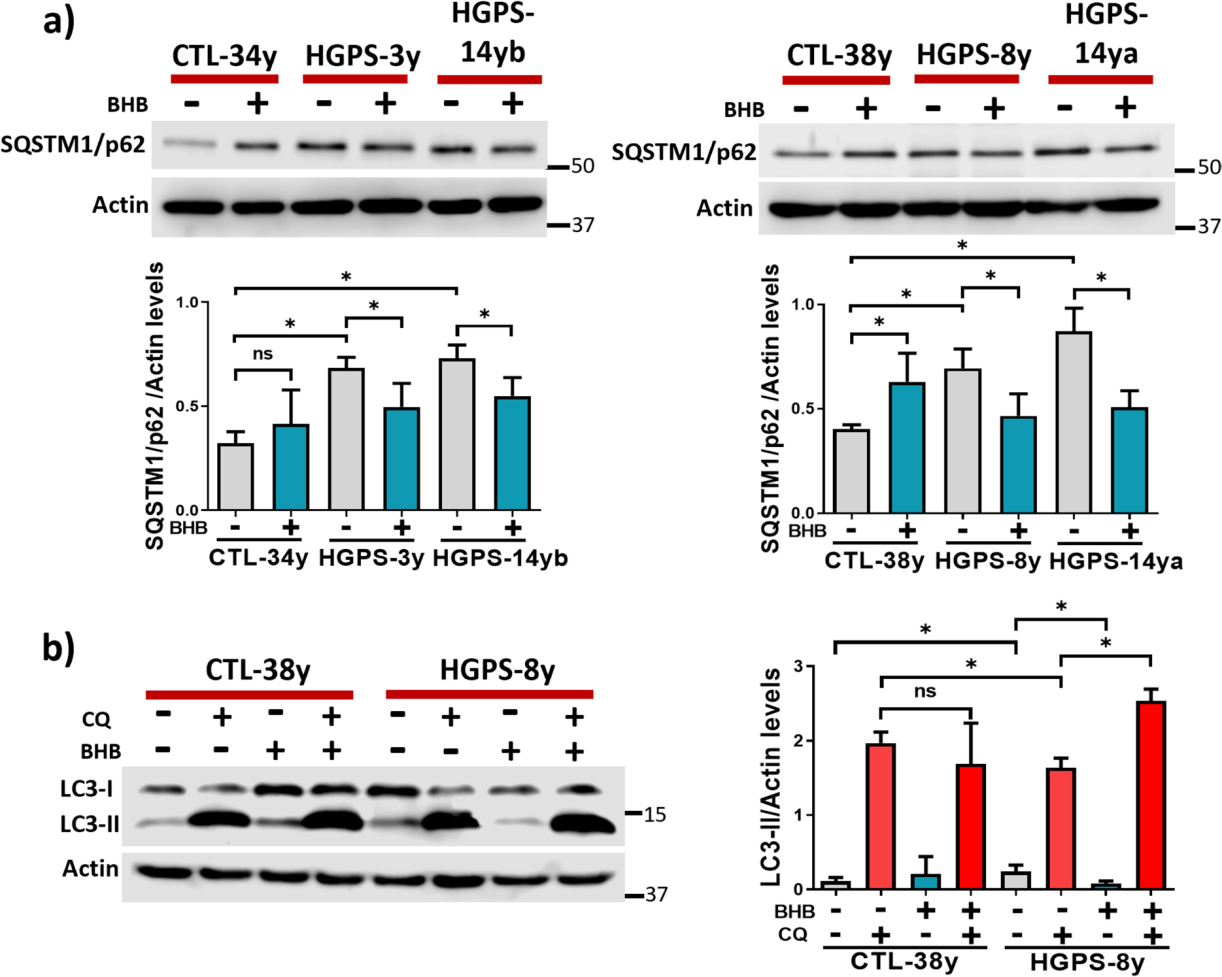
BHB stimulates the autophagic flux in HGPS. **a** Representative WB of SQSTM/p62 and actin (loading control) from CTL-34y, HGPS-3y, HGPS-14yb, CTL-38y, HGPS-8y, and HGPS-14ya cell cultures treated or not with 3 mM BHB for 72 h. The graph shows the relative protein content of SQSTM/p62 represented as mean ± SD from three independent experiments.** b** *Left panel*, representative WB of LC3 and actin (loading control) from CTL-38y and HGPS-8y cell cultures treated or not with 3 mM BHB for 72 h and with CQ at 50 μM during the last 12 h of BHB treatment. The graph corresponds to the relative protein content of LC3-II represented as the mean ± SD from three independent experiments. Statistical differences were calculated using the unpaired *t*-test; *p < 0.05; ns, no statistical significance

Several therapeutical strategies for HGPS revealed that progerin clearance can be achieved by autophagy induction; hence, we sought to test whether autophagy is related to the BHB effect on progerin protein decline. As mentioned above, autophagy is impaired in HGPS contributing to the senescent phenotype; thereby, we first assessed whether BHB can induce autophagy. With this purpose, we evaluated the SQSTM1/p62 protein levels, which are elevated when autophagic degradation is impaired and reduced when the autophagic flux is active. We found that SQSTM1/p62 protein levels were elevated in all HGPS fibroblast cultures compared to CTL cultures (Fig. 6a), indicative of autophagy degradation impairment. SQSTM1/p62 content was significantly decreased after BHB treatment in all cases suggesting an improved autophagic degradation (Fig. 6a). To confirm this observation, we assessed the autophagy flux by determination of LC3-II levels (indicative of autophagosome formation) in BHB-treated and non-treated HGPS-8y fibroblasts, using the autophagy inhibitor chloroquine (CQ) that blocks autophagosome-lysosome fusion and leads to autophagosome accumulation [30]. As expected, we observed a statistically significant increase in LC3-II levels when healthy CTL-38y and HGPS-8y cultures were treated with CQ; however, we found a subtle decrease of LC3-II in HGPS-8y fibroblasts as compared to CLT-38y cultures, suggesting lower autophagosome accumulation. When cells were co-treated with CQ and BHB, a significant elevation of LC3-II content was observed in HGPS-8y fibroblasts (Fig. 6b). Altogether, these data indicate that the autophagy flux is diminished in HGPS and improved after BHB treatment, as previously suggested [28].

**Fig. 7:**
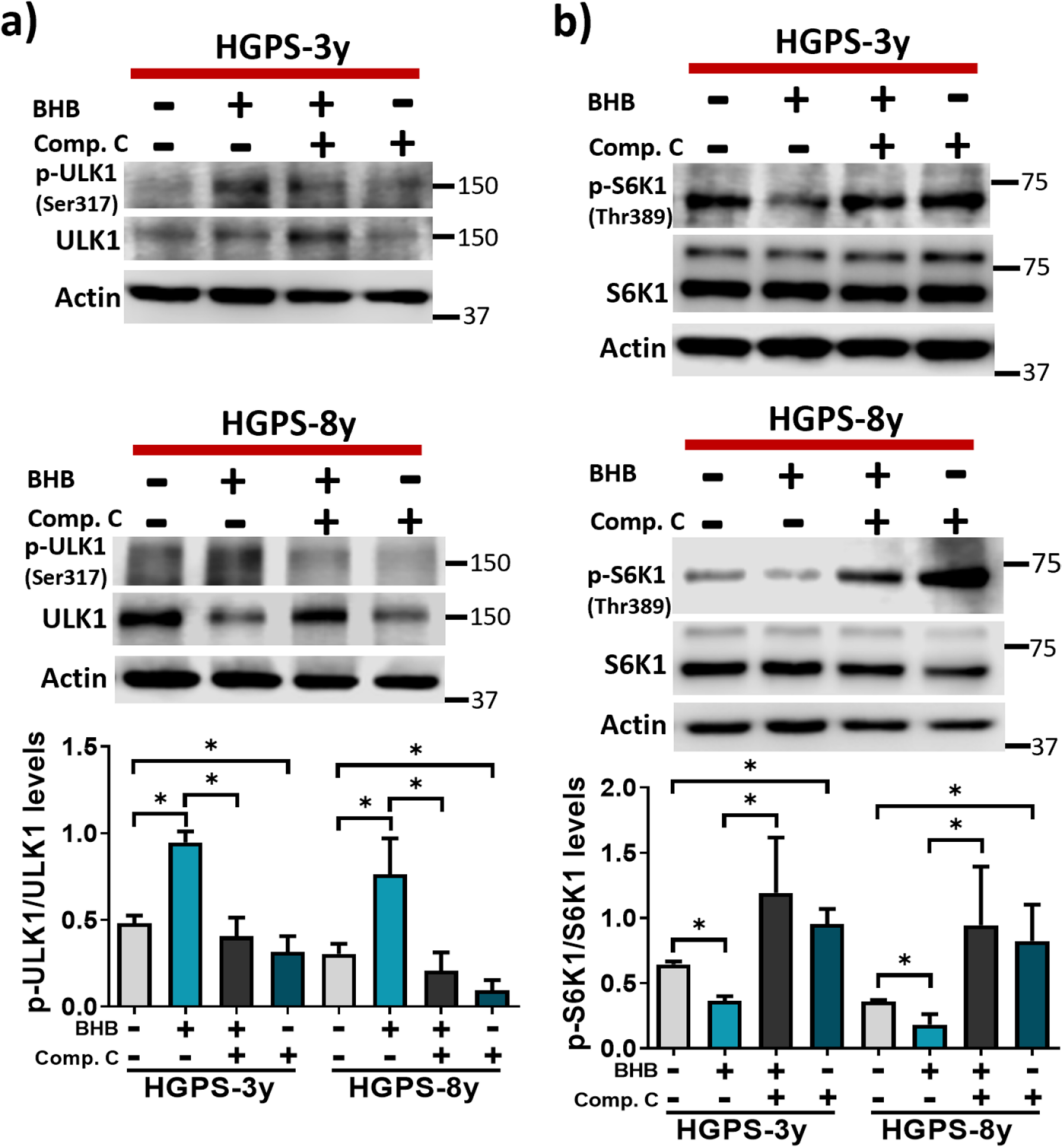
BHB induces AMPK activity. **a**, **b** *Upper panel*, representative WB of p-ULK1, ULK1, p-S6K1, S6K1, and actin (loading control) from HGPS-3y and HGPS-8y cell cultures treated or not with 3 mM of BHB and 5 μM of compound C for 24 h. *Graphs* show the relative protein abundance of pULK1 and pS6K1 normalized to total ULK1 and S6K, respectively. Data represent the mean ± SD of three independent experiments. Statistical differences were calculated using the unpaired *t*-test; *p < 0.05

Then, we sought to test whether autophagy is responsible for progerin diminishing by BHB. Briefly, we treated HGPS-3y and HGPS-8y fibroblasts for 1 day with BHB in combination with the autophagy inhibitors; bafilomycin A1 (BafA1), an inhibitor of the lysosomal H^+^-ATPase and the autophagosome-lysosome fusion [30]; and compound C (Comp. C), an inhibitor of AMPK (the inductor of autophagy initiation) [55–60]. With this purpose, we determined the phosphorylation levels of ULK1 (Ser317) by AMPK, and mTORC1 activity through the phosphorylation of its direct target, the ribosomal protein S6 kinase beta-1 (S6K1) phosphorylated at Thr389 [61]. AMPK phosphorylates ULK1 at serine 317 to promote autophagy initiation; thus, we speculated that BHB will increase p-ULK1 (ser317), and compound C will diminish this phosphorylation. As expected, BHB elevated p-ULK1 (ser317), and this was reversed by compound C (Fig. 7a), suggesting that BHB induces AMPK activity. If this is true, AMPK activation by BHB in HGPS will lead to mTORC1 inhibition and decreased p-S6K1 (Thr389) levels. Consistently, we observed that BHB treatment diminished p-S6K1 (Thr389) levels and compound C co-treatment reversed this effect (Fig. 7b), confirming that BHB inhibits mTORC1 activity via AMPK activation. We also evaluated SQSTM/p62 and LC3-II content after 1-day co-treatment with BHB and compound C in HGPS-3y and HGPS-8y fibroblast. We found that SQSTM/p62 and LC3-II decreased after 1-day BHB treatment, but when co-treated with compound C, SQSTM/p62 and LC3-II protein levels augmented in both cell cultures (Fig. S4a). We also observed that compound C alone elevated the levels of LC3-II in HGPS cells (Fig. S4a), suggesting the stimulation of autophagy, which has been previously reported in U251 glioma cells [62], however, SQSTM/p62 content was also augmented suggesting that autophagy stimulation by this inhibitor is not functional.

In order to confirm that autophagy induction by BHB is AMPK-dependent, we used the ULK1/2 inhibitor, SBI, which also inhibits AMPK activity [63, 64]. As shown in Fig. S4b, SBI reversed the effect of BHB on SQSTM/p62 as observed with compound C, suggesting it blocks the BHB-induced stimulation of the autophagic flux. The effect of SBI on LC3-II was more variable, and no effect of SBI on BHB-induced decrease of LC3-II was observed. However, LC3-II levels tended to decrease when SBI was incubated alone. Together, these data suggest that BHB stimulates autophagy via the AMPK-mTOR-ULK1 pathway.

**Fig. 8:**
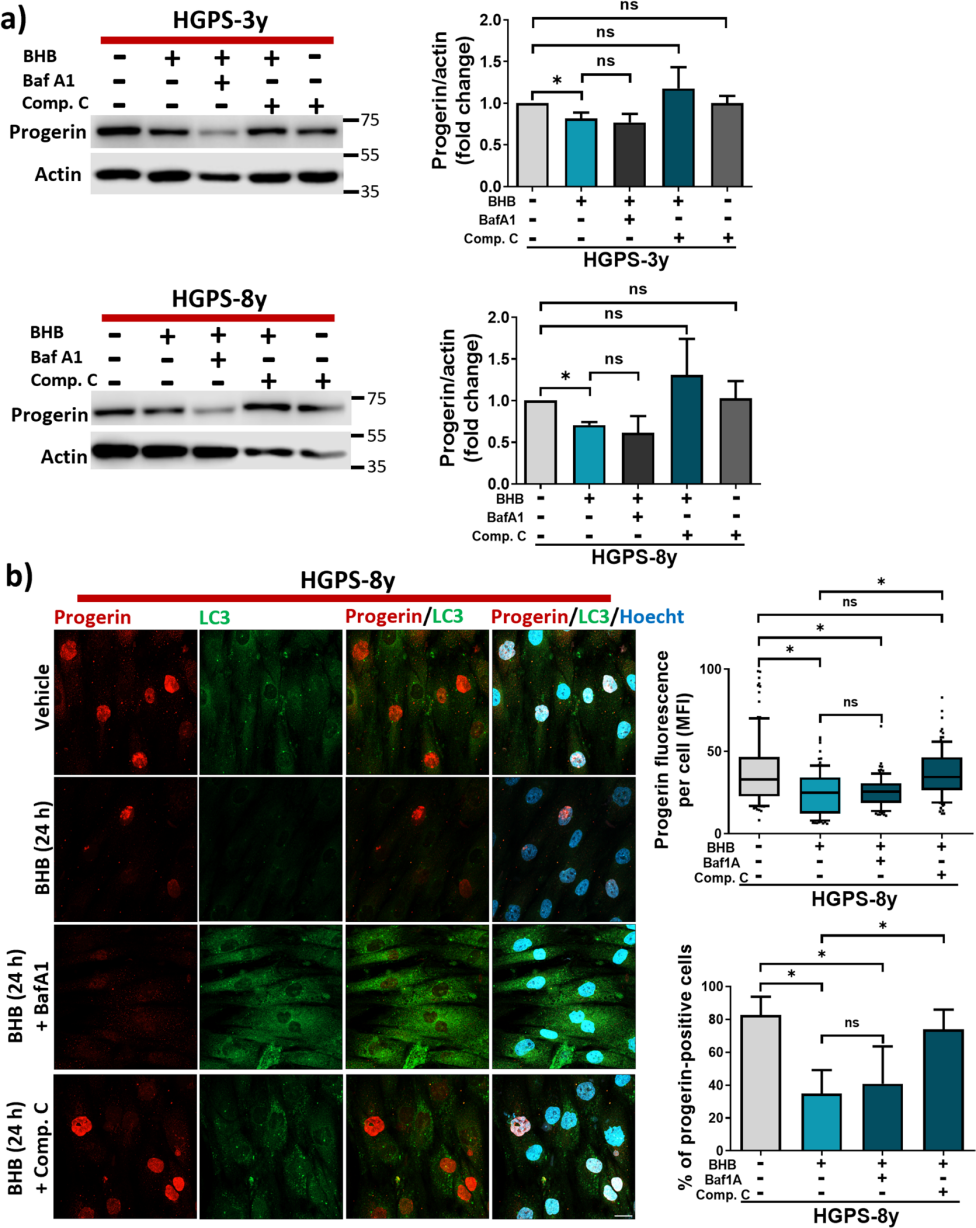
Compound C prevents BHB-induced progerin reduction in HGPS.** a** *Left panel*, WB of progerin and actin (loading control) in HGPS cell cultures treated or not with 3 mM BHB for 24 h in combination with 200 nM BafA1 or 5 μM compound C. *Right panel*, graphs show the relative progerin abundance expressed as mean ± SD from three independent experiments. Statistical differences were calculated using the unpaired *t*-test; *p < 0.05; ns, no statistical significance. The mean value obtained in vehicle-treated HGPS cell cultures was set at 1, and the mean value of the treated HGPS was normalized to this value. **b** *Left panel*, IF of progerin in HGPS-8y cell cultures treated with 3 mM of BHB in combination with 200 nM BafA1, 5 μM compound C, or vehicle for 24 h. Nuclei were stained with Hoechst; scale bar 10 µm. *Right panel*, graph shows the mean progerin immunofluorescence intensity (MFI) per cell from three independent experiments. Data are shown in a box and whiskers graph (percentile 10–90, *n* = 100 cells per condition), and the bottom graph shows the percent of progerin-positive cells as mean ± SD (*n* = 100 cells per condition). Statistical differences were calculated using the nonparametric Mann–Whitney test and unpaired *t*-test, respectively; *p < 0.05; ns, no statistical significance

**Fig. 9:**
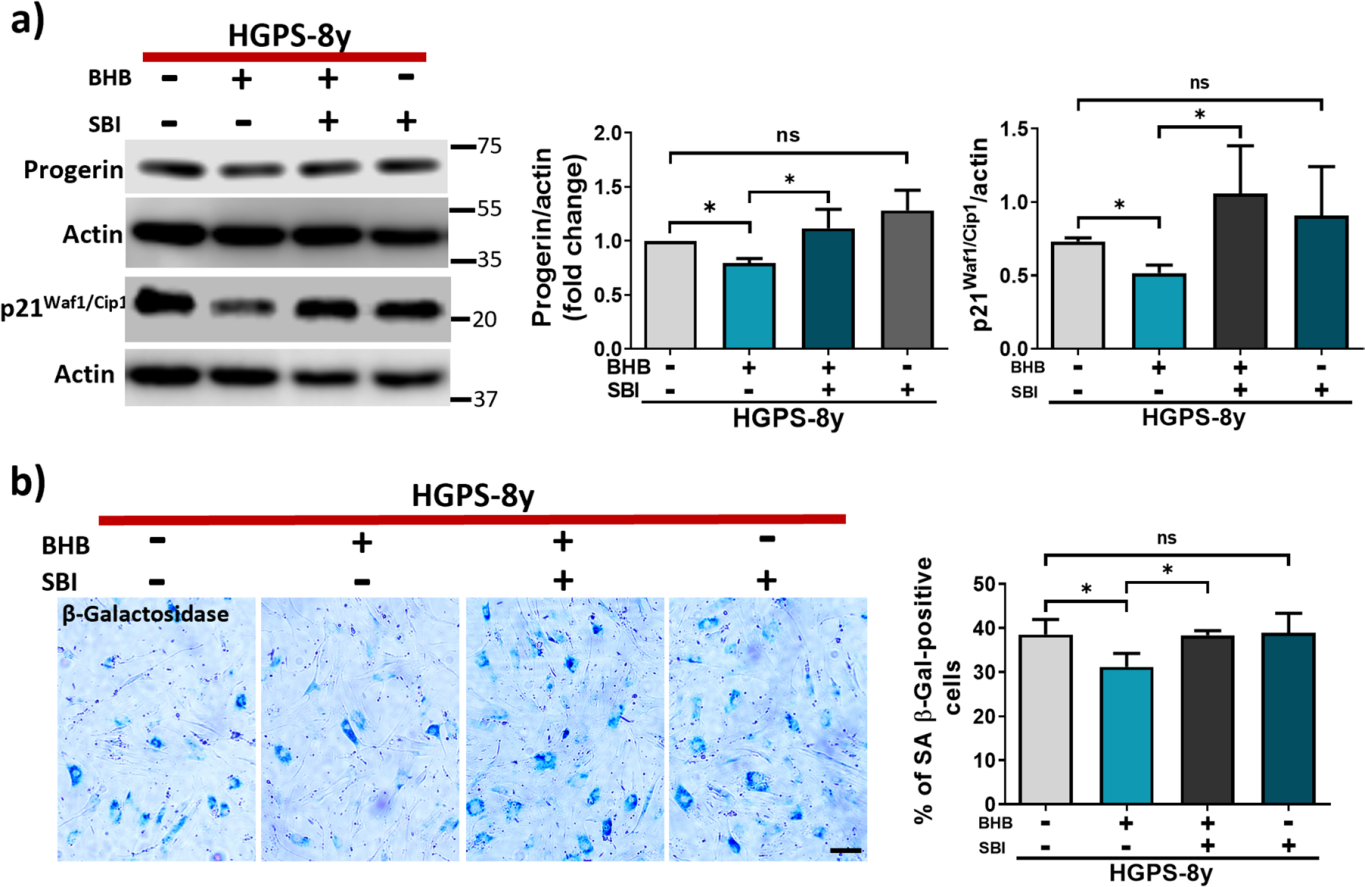
**a** *Left panel*, representative WB showing progerin and p21^Waf1/Cip1^ protein abundance in HGPS-8y fibroblasts cell cultures treated or not with BHB 3 mM and SBI 10 μM for 24 h. Actin was used as a loading control. *Right panel*, graph shows progerin and p21^Waf1/Cip1^ protein abundances plotted as mean ± SD (three independent replicates per group). Statistical differences were calculated using the unpaired t-test test; *p < 0.05; ns, no statistical significance. **b** *Left panel*, representative images of SA β-Gal-positive cells (blue) in treated HGPS-8y fibroblast cultures (panel a scale bar = 60 μM). *Right panel*, the percentage of SA β-Gal-positive was plotted as mean ± SD (*n* = 300 cells per condition from three independent replicates per group)

Then protein levels of progerin were assessed in the presence of compound C, BafA1, and SBI. We found that 1-day treatment with BHB reduces progerin as previously observed (Fig. 1b), and this reduction was reversed by compound C, but not with BafA1 (Fig. 8a, b). The incubation of compound C alone induced no change in progerin content (Fig. 8a). Then, we aimed to corroborate these findings, using SBI on BHB-induced progerin decline. As observed with compound C, SBI also prevented the BHB-induced progerin decrease, and progerin remained unchanged when SBI was incubated alone (Fig. 9a). BafA1 reversed the decrease in SQSTM1/p62 induced by BHB leading to its accumulation (Fig. S4c). Therefore, inhibiting autophagic degradation by BafA1 antagonizes the effect of BHB on the autophagic flux. However, BafA1 did not block the BHB-induced reduction of progerin (Fig. 8a, b), suggesting an additional effect of this compound on progerin content.

Since SBI was able to impair autophagy degradation and prevent progerin clearance upon BHB treatment, we tested whether SBI would be able to prevent the anti-senescent effect of BHB as well. To test this hypothesis, the number of SA β-Gal-positive cells and p21 protein levels were evaluated in HGPS-8y fibroblast treated with BHB and SBI for 24 h. It was observed that SA β-Gal-positive cells and p21 protein levels diminished after 24 h BHB treatment. Consistently, this was prevented after SBI-BHB co-treatment (Fig. 9a, b). Treatment with SBI alone had no effect (Fig. 9a, b). This data confirmed that the BHB anti-senescent effect relies on the clearance of progerin, which is mediated by autophagy induction.

### Comparison of BHB anti-senescent effect with that of MG132 and rapamycin

**Fig. 10:**
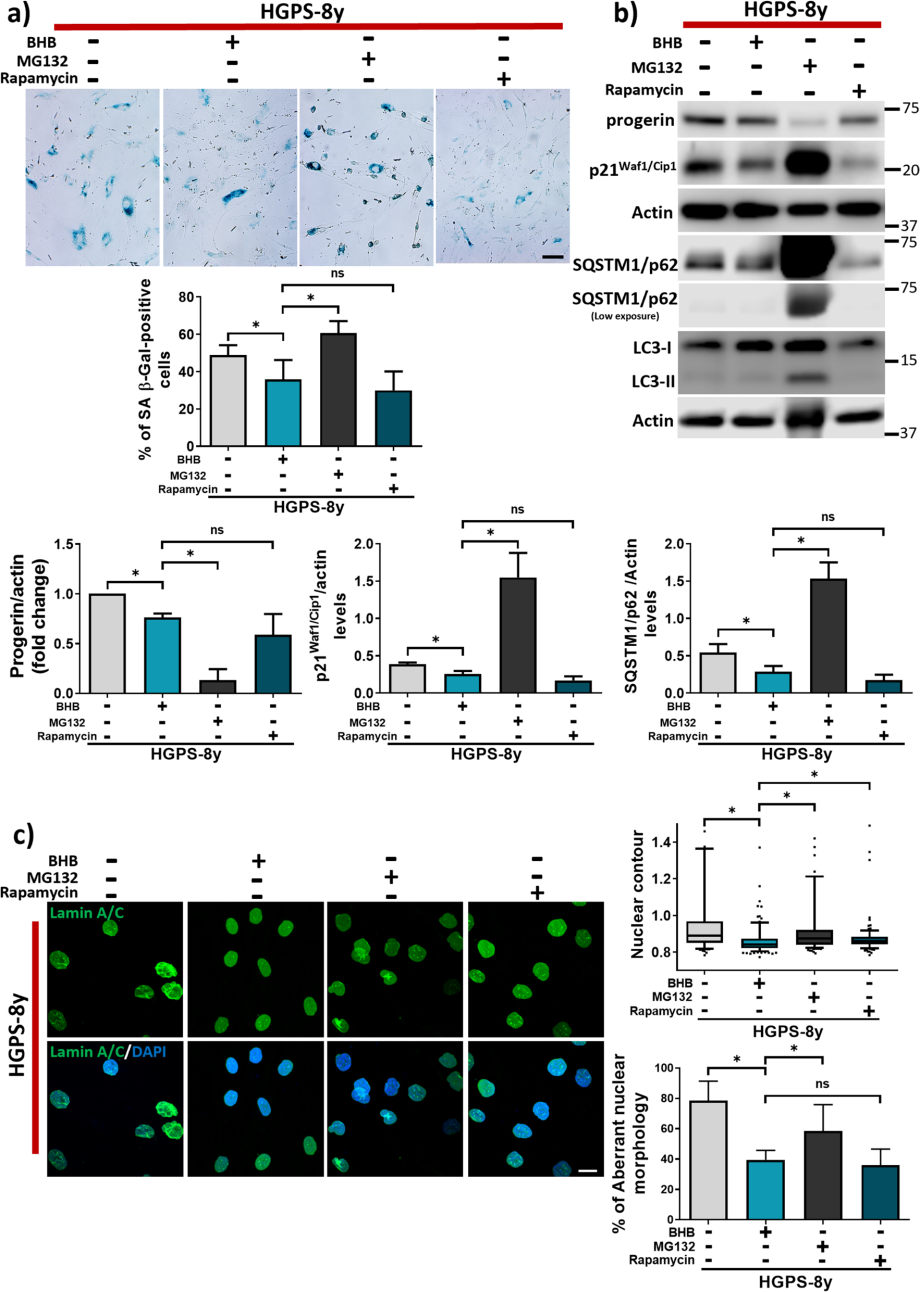
**a** *Upper panel*, representative images of SA β-Gal-positive cells (blue) in HGPS-8y fibroblast cultures treated or not with BHB 3 mM, MG132 0.5 µM, and rapamycin 1 µM by 72 h (scale bar = 60 μM). *Lower panel*, the percentage of SA β-Gal-positive cells was plotted as mean ± SD (*n* = 300 cells per condition from three independent replicates per group). **b** *Upper panel*, representative WB showing progerin, p21^Waf1/Cip1^, SQSTM1/p62, and LC3-II protein abundance in HGPS-8y fibroblast cell cultures. Actin was used as a loading control. *Lower panel*, graph shows progerin, p21^Waf1/Cip1^, and SQSTM1/p62 protein abundances plotted as mean ± SD (three independent replicates per group). Statistical differences were calculated using the unpaired *t*-test test; *p < 0.05; ns, no statistical significance. **c** *Left panel*, representative images of the comparison between BHB, MG132, and rapamycin treatments on nuclear morphology by IF of Lamin A/C on HGPS-8y fibroblast. Nuclei were stained with DAPI; scale bar 20 µm. *Right panel*, nuclear contour data was plotted in a box and whisker graph (percentile 10–90, *n* = 100 cells per condition). Significant differences were obtained using the nonparametric Mann–Whitney test; *p < 0.05; ns, no statistical significance. Qualitative analysis of nuclear aberrant morphology. The percentage of aberrant nuclei was plotted and shown as the mean ± SD (*n* = 100 cells per condition). Statistical differences were calculated using the unpaired *t*-test test; *p < 0.05

Finally, we compared the effect of BHB with two molecules capable of inducing autophagy that have been previously studied for HGPS treatment: MG132 and rapamycin. Thus, the effects of these compounds on HGPS senescent phenotype and autophagy were compared with those of BHB. With this purpose, the number of SA β-Gal-positive cells, progerin and p21 protein abundance, nuclear morphology, and SQSTM1/p62 and LC3-II protein levels were determined in HGPS fibroblasts individually treated with BHB 3 mM, MG132 0.5 µM [38, 65], and rapamycin 1 µM [37, 66, 67] for 72 h. We found that the effect of BHB was similar to that of rapamycin, as both molecules induced progerin clearance, reduced the number of SA β-Gal-positive cells and the abundance of p21, improved the nuclear morphology, and diminished SQSTM1/p62 protein levels, indicating that autophagy was improved and senescence decreased after the treatment (Fig. 10 and Fig. S5). In contrast, MG132 induced progerin clearance but failed to induce autophagic degradation and decrease senescence markers, as observed by augmented SQSTM1/p62 and p21 protein levels. Also, MG132 failed to decrease SA β-Gal-positive cells (Fig. 10 and Fig. S5). Altogether, these results indicate that BHB treatment alleviates the senescence phenotype in HGPS, similarly to rapamycin, through progerin clearance via AMPK-mTORC1-ULK1 autophagy activation.

## Discussion

In this study, we demonstrate for the first time the anti-senescence effect of BHB on human dermal fibroblast cultures from HGPS patients. BHB exposure decreased progerin content in all HGPS fibroblast cultures assessed, alleviating several of the downstream progerin-induced cellular alterations implicated in senescence, such as nuclear and nucleolar morphology abnormalities [2, 3]. So far, no evidence has been reported about the effect of BHB on nuclear and nucleolar architecture. Here, we have observed that BHB reduces progerin and Lamin A levels and recovers Lamin B1 content in HGPS fibroblast cells. The latter is in line with a previous report demonstrating a BHB-dependent elevation of Lamin B1 in vascular senescence [17]. In the case of Lamin A/C and progerin levels, to our knowledge, no data has been previously reported. However, direct β-hydroxybutyrylation of Lamin A/C, as well as other relevant components of the nuclear lamina such as Lamin B2 and lamina-associated polypeptide 2, has been recently reported as a post-translational modification in a proteomic analysis in HEK293 cells and senescent mice hearts [68–70]. Regarding the nucleolus, in the PLC-PRF-5 epithelial liver cell line, BHB drives nucleophosmin/B23 upregulation through the enrichment of H3K9bhb [71]; moreover, nucleophosmin/B23 can be directly β-hydroxybutyrylated [68]. This data suggests a nuclear and nucleolar architecture modulation by BHB. Further studies are needed to elucidate this regulation in detail.

The anti-senescence effect of BHB has been reported previously in several models including human aortic smooth muscle cells and mice aorta, where Lamin B1 is elevated and OCT4 mRNA is stabilized via heterogeneous nuclear ribonucleoprotein A1 (hnRNP A1) [17]; in rat cartilage and human osteoarthritic chondrocytes, BHB reduces senescence by PTEN stabilization via hnRNP A1 [18]; in mice diabetic kidney disease, BHB alleviates senescence by reinforcing Nrf2 response via GSK3ß inhibition [72]; and in rat hepatic cells, BHB alleviates senescence via AMPK-mTOR autophagy induction [42]. However, the effect of BHB on cellular premature senescence associated with progeria has not been studied before. We successfully replicated the antisenescence effect of BHB in HGPS fibroblasts, as we observed a decrease in SAβ-Gal-positive cells and DNA damage foci, augmented content of Lamin B1, p21^Waf1/Cip1^ and p16^CDKN2A^ decreased abundance, cellular area reduction, and recovery of the heterochromatin H3K9me3 mark after BHB treatment.

The present results suggest that BHB alleviates autophagy impairment, another hallmark of aging [8]. Increased levels of SQSTM/p62 were observed in HGPS fibroblast cultures derived from four different donors relative to cultures from normal subjects, suggesting decreased autophagy degradation. In the case of LC3-II, we observed very low levels in normal and HGPS cultures, but in the presence of CQ, HGPS-8y showed less accumulation of LC3-II relative to normal fibroblasts, suggesting a diminished autophagic flux. Remarkably, BHB reversed these parameters, leading to the conclusion that BHB stimulates autophagy in HGPS. This is consistent with many studies converging on BHB stimulation of autophagy by diverse mechanisms such as the stimulation of the AMPK-mTOR [42] and the SIRT2-FOXO1-FOXO3a pathways, lysosomal biogenesis [22, 23], autophagy flux stimulation [25, 28], induction of chaperone-mediated autophagy (CMA) by substrate oxidation [24], and mitophagy induction [23, 73].

Autophagy stimulation by BHB has been related to the degradation of specific altered proteins associated with neurodegeneration such as the amyloid-β precursor protein [74] and Tau/pTau [75], but this has not been tested for progerin elimination. In the present study, BHB autophagy induction was associated with decreased progerin levels in HGPS cells. According to the present results, this effect is mediated by increased AMPK activity after BHB treatment, as indicated by the elevation of its downstream target, p-ULK (Ser317). Furthermore, mTOR inhibition by BHB treatment is suggested by the decreased phosphorylation of its downstream target, p-S6K (Thr389). These results are consistent with previous reports that indicate the BHB-dependent activation of AMPK and autophagy [23, 44, 76, 77]. Moreover, these BHB-induced effects, as well as the anti-senescent effect and progerin clearance, were blocked by compound C and SBI, suggesting an AMPK-ULK1-dependent effect of BHB on senescence. In line with these findings, it has been reported that the AMPK activator 991 induces autophagy as well as autophagy-dependent progerin decline in HGPS cells, driving senescence alleviation [31].

When autophagic degradation was inhibited with BafA1, the decline in progerin levels induced by BHB was not restored. This effect might be related to the previously reported senolytic action (that is, the elimination of senescent cells) of BafA1, in induced senescent cells [78], which might prevent progerin accumulation. More studies on the BafA1 senolytic effect on HGPS and how it correlates with progerin protein abundance are needed to clarify this subject.

Regarding the comparison of BHB with MG132 and rapamycin, a similar anti-senescence effect was observed between BHB and rapamycin. Moreover, both molecules reduced senescence markers and induced autophagy and progerin clearance. These data are relevant, since rapamycin is one of the most effective candidate molecules against senescence, according to in vitro and in vivo studies [79]. Therefore, we speculate that BHB can have potential for aging therapeutics, resulting in an alternative treatment to rapamycin precluding its immunosuppressant effect [80, 81]. More studies are needed to assess this matter. Considering the multiple beneficial effects of BHB reported so far [14, 82–84], it is worth to determine whether other actions of BHB are related to its anti-senescent effect. On the other hand, a decrease in progerin abundance and an increase in LC3-II levels were observed after MG132 treatment, as previously reported [38, 65]. However, in the present experimental conditions, it failed to reduce senescence, and apparently, it also failed to improve the autophagic flux, as observed by the notable accumulation of SQSTM1/p62 protein levels in the presence of MG132.

This work provides evidence for the first time about the potential therapeutic effect of BHB in HGPS-senescent phenotype via autophagy-dependent progerin clearance, opening the door for a new therapeutic approach. In HGPS and normal aging hearts, ketone metabolism is enhanced; therefore, BHB becomes an important energy source [85–87]. Indeed, treatment with the ketone ester or BHB alleviates vascular senescence [17] and heart failure in mice and rats [73, 84, 88, 89]. This is relevant to HGPS, as heart failure is the main cause of mortality in these patients. In conclusion, the present data suggests BHB administration as a potential treatment for HGPS. More in vivo studies in murine models are needed to further confirm the present findings.

## Supplementary Information

Below is the link to the electronic supplementary material.

Supplementary file1 (DOCX 6.37 KB)

## Abbreviations

BHB: D-β-hydroxybutyrate
HGPS: Hutchinson-Gilford progeria syndrome
IF: Immunofluorescence
WB: Western blot
*LMNA*: Lamin A/C gene
AMPK: 5′Adenosine monophosphate-activated protein kinase
ZMPSTE24: Zinc metallopeptidase STE24
FTI: Farnesyl transferase inhibitor
OCT4: Octamer-binding transcription factor 4
PTEN: Phosphatase and tensin homolog
LC3: Microtubule-associated protein 1A/1B-light chain 3
p62/SQSTM: P62/sequestosome 1
mTOR: Mammalian target of rapamycin
ULK1: Unc-51-like kinase 1
MFI: Mean fluorescence intensity
S6K1: Ribosomal protein S6 kinase beta-1
