# Supplementary material for: The anti-senescence effect of D-β-hydroxybutyrate in Hutchinson-Gilford progeria syndrome involves progerin clearance by the activation of the AMPK-mTOR-autophagy pathway
Source: GeroScience. 2025 Jan 16;47(3):3849–71. doi: 10.1007/s11357-024-01501-9 (PMC12181494; doi:10.1007/s11357-024-01501-9)
Supplement: Supplementary file 1 — Supplementary file1 (DOCX 6.37 KB) [file 11357_2024_1501_MOESM1_ESM.docx]

**Supplementary Information for publication of**

**The anti-senescence effect of D-β-hydroxybutyrate in Hutchinson-Gilford progeria syndrome involves progerin clearance by the activation of the AMPK-mTOR-autophagy pathway**

GeroScience

Feliciano Monterrubio-Ledezma^1,3^, Ashley Salcido-Gómez^1^, Tania Zavaleta-Vásquez^2^, Fernando Navarro-García^3^, Bulmaro Cisneros^2^, Lourdes Massieu^1, *^.

^*^ To whom correspondence should be addressed (**lmassieu@ifc.unam.mx**).

**This file includes:**

Figs. S1 to S5

**
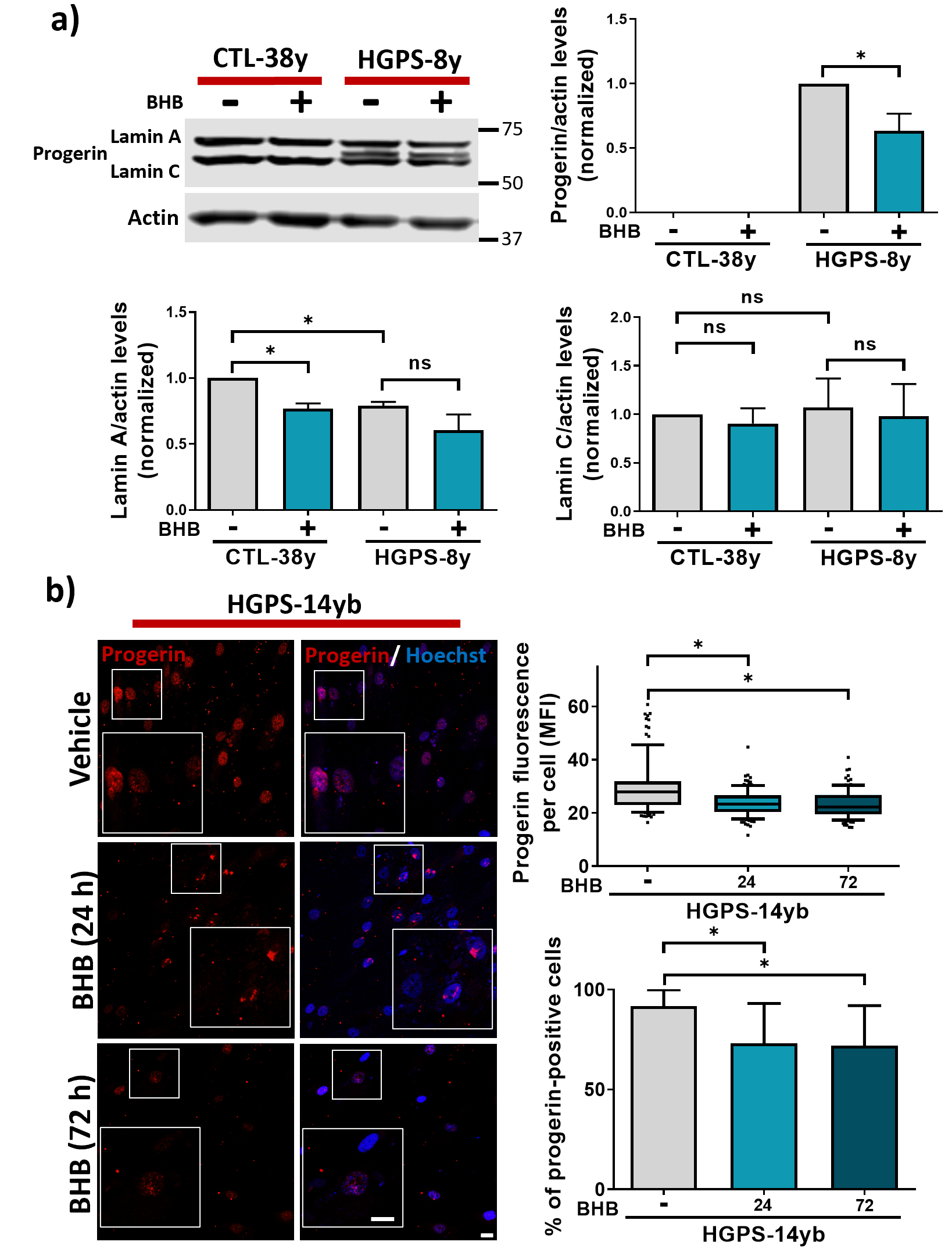
**

**Figure S1.** **a)** *Upper panel*. WB of CTL-38y and HGPS-8y cell cultures treated or not with 3 mM of BHB for 72 h using anti-Lamin A/C and anti-actin (loading control) antibodies. *Right and* *lower panel*. The graphs indicate the relative protein abundance of progerin, lamin A and lamin C expressed as the mean ± SD from three independent experiments. Statistical differences were calculated using the unpaired t-test; * p < 0.05; ns: no statistical significance. **b)** *Left*. IF of HGPS-14yb cell cultures treated or not with 3 mM of BHB for 24 and 72 h using the anti-progerin antibody. Nuclei were stained with Hoechst; scale bar 20 µm. *Right*. The mean progerin immunofluorescence intensity (MFI) per cell was quantified and data from three independent experiments are shown in a box and whiskers graph and (percentile: 10–90, n = 100 cells per condition) the graph below shows the percent of cells with progerin, expressed as mean ± SD (n = 100 cells per condition). Statistical differences were calculated using the nonparametric Mann–Whitney test and unpaired t-test, respectively; * p < 0.05.

**
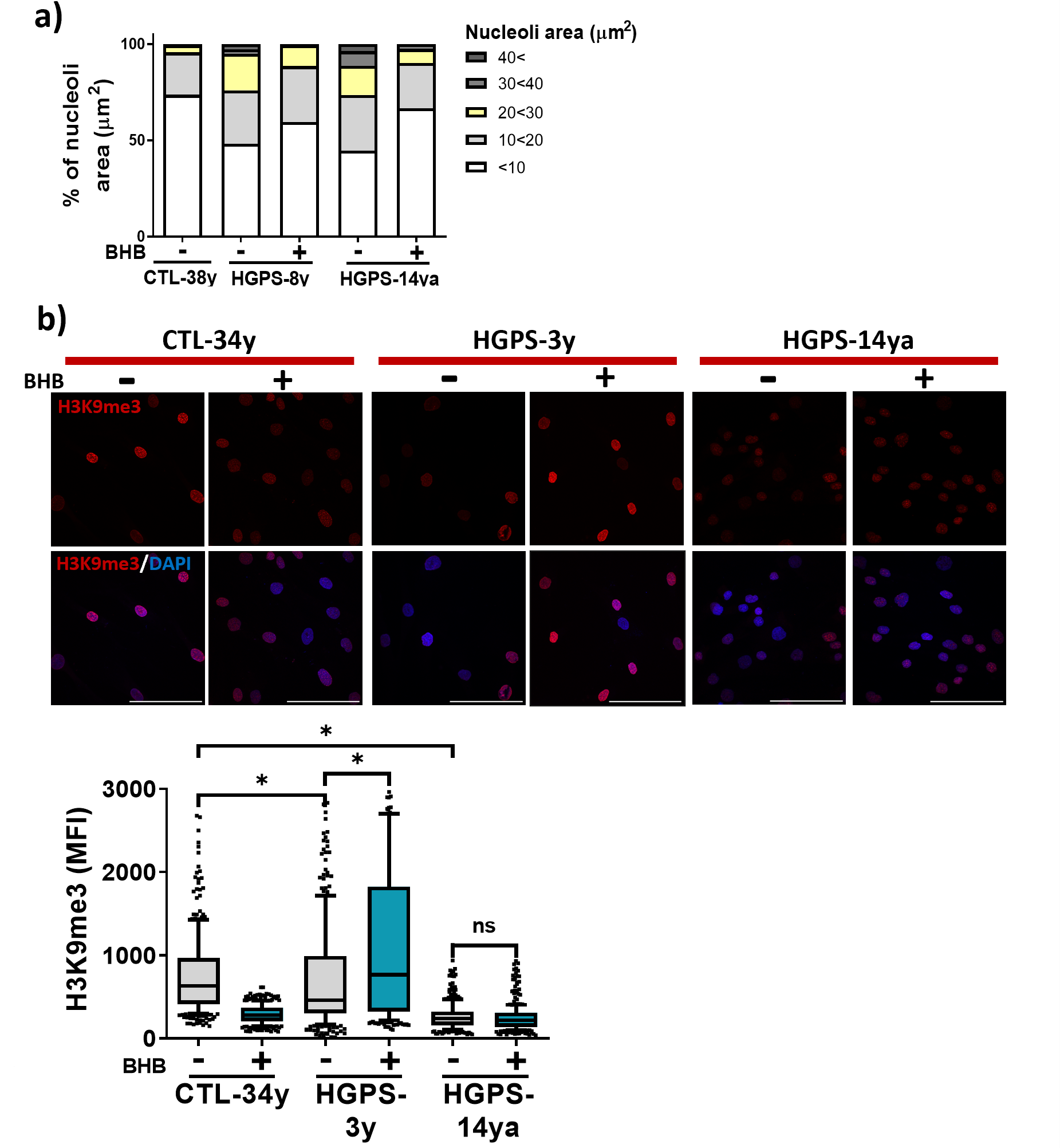
**

**Figure S2. a)** Area per nucleoli from figure 3d data was clustered in five categories and expressed as percentage of nucleoli area. **b)** *Upper panel*. IF of CTL and HGPS cell cultures treated or not with 3 mM of BHB for 72 h using the anti-H3K9me3 antibody. Nuclei were stained with DAPI; scale bar 50 µm. *Lower panel*. The mean H3K9me3 fluorescence intensity (MFI) per cell ± SD was quantified and data from three independent experiments are shown in a box and whiskers graph (percentile: 10–90, n = 300 cells per condition). Statistical differences were calculated using the nonparametric Mann–Whitney test; * p < 0.05; ns: no statistical significance.

**
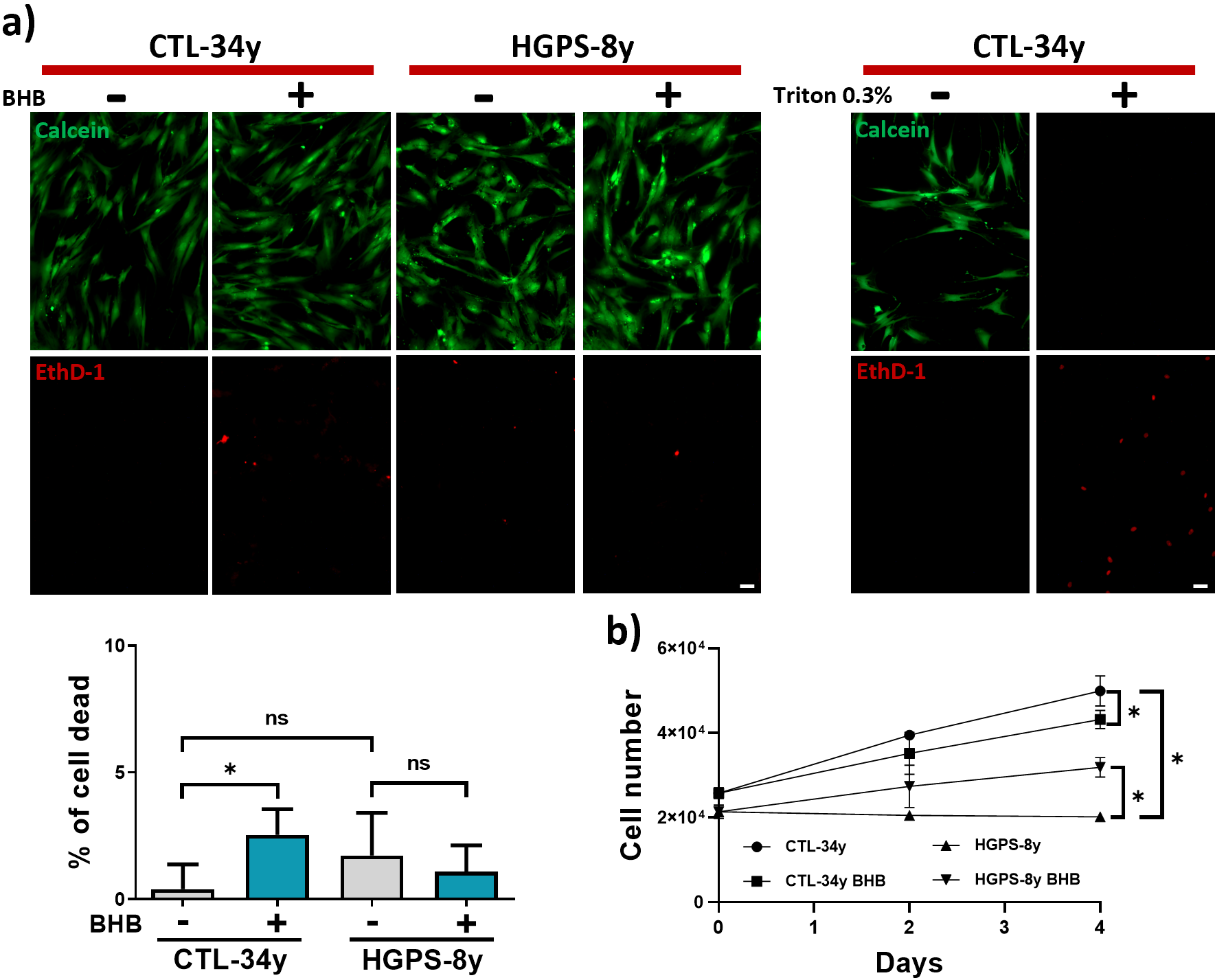
**

**Figure S3**. **a)** Effect of BHB on cell viability and proliferation in HGPS-8y and CTL-34y fibroblasts. Cells were treated with BHB for 72 h and cell viability assessed by the Live/Dead assay, calcein-AM (green, living cells) and EthD-1 (red, dead cells). *Upper panel*. Representative images are shown, scale bar = 25 µm. *Lower panel*. The percentage of cell dead was calculated and expressed as mean ± SD (n = 300 cells per condition). b) To determine cell proliferation cells were harvested at 0, 48 and 96 h, and the number of cells counted using a Neubauer chamber. Statistical differences were calculated using the unpaired two-way ANOVA test; * p < 0.05; ns: no significant.


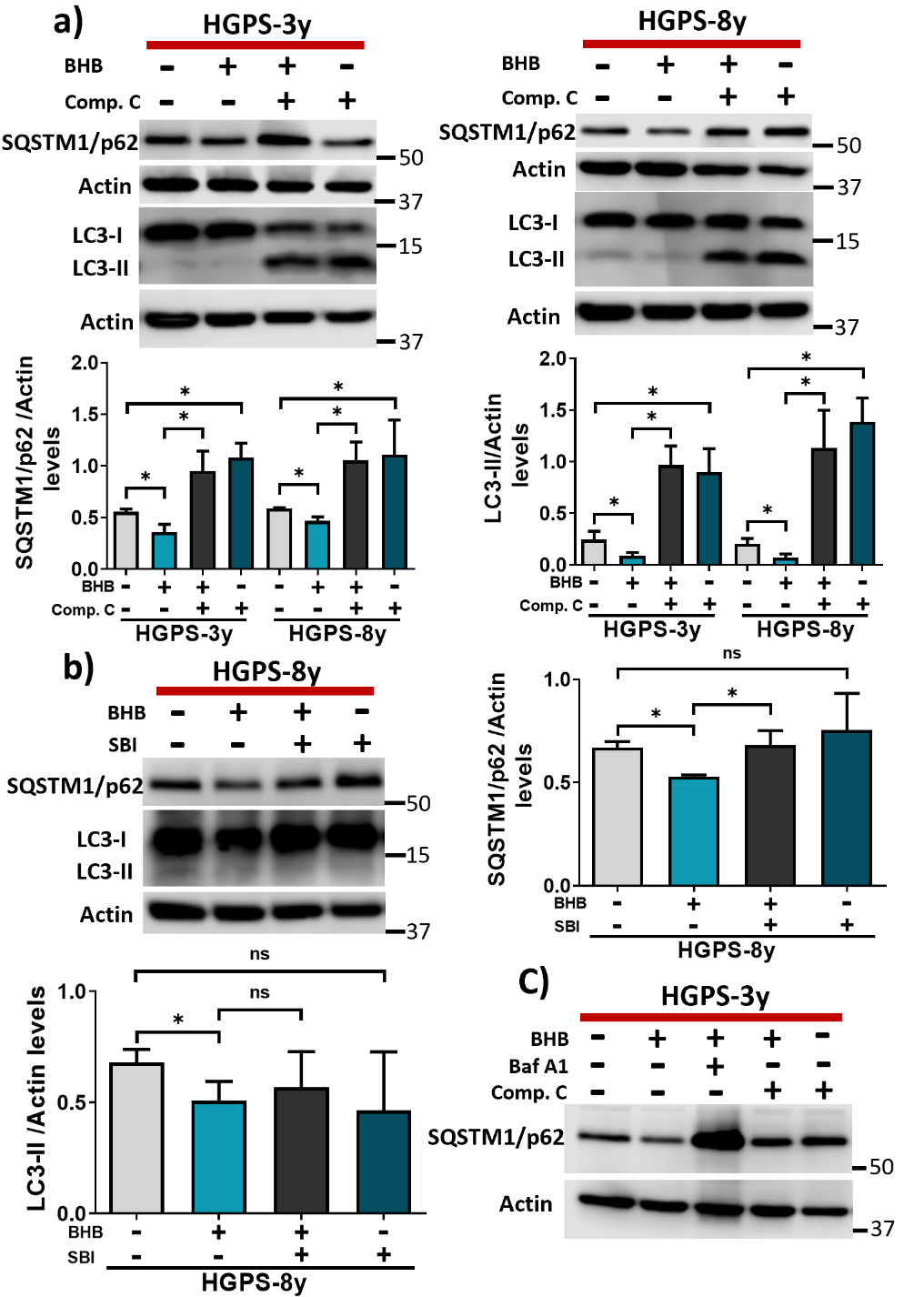


**Figure S4. a)** *Upper panel.* WB of HGPS-3y and -8y cell cultures treated or not with 3 mM BHB and 5 μM Compound C for 24 h were using anti-SQSTM/p62, LC3 and anti-actin (loading control) antibodies. *Lower panel*. The graphs correspond to the relative protein content expressed as the mean ± SD from three independent experiments. **b)** *Upper panel*. WB of HGPS-3y and -8y cell cultures treated or not with 3 mM BHB and 10 μM SBI for 24 h using anti-SQSTM1/p62, LC3 and anti-actin (loading control) antibodies. *Lower panel*. The graphs correspond to the relative protein content expressed as the mean ± SD from three independent experiments. Statistical differences were calculated using the unpaired t-test; * p < 0.05. **c)** WB showing the effect of BafA1 on SQSTM1/p62 protein levels in HGPS-3y cultures treated with BHB using anti-SQSTM1/p62 and anti-actin (loading control) antibodies.

**Figure S5 a)** *Left panel*. Representative images of SA β-Gal positive-cells (blue) in HGPS-14yb fibroblast cultures treated with BHB 3mM, MG132 0.5 µM or rapamycin 1 µM for 72 h (scale bar = 60 μM). *Right panel*. Percentage of SA β-Gal positive-cells plotted as mean ± SD (n = 300 cells per condition from three independent replicates per group). **b)** Comparison between BHB, MG132 and rapamycin treatments on nuclear morphology by IF of Lamin A/C on HGPS-3y fibroblast. Nuclei were stained with DAPI; scale bar 20 µm. *Lower panel.* *Left*. Nuclear contour was determinate using NII plug-in from three independent experiments per group. Data were plotted in a box and whiskers graph (percentile: 10–90, n = 100 cells per condition). Significant differences were obtained using the nonparametric Mann–Whitney test; * p < 0.05; ns: no statistical significance. *Right*. Qualitative analysis of nuclear aberrant morphology. The percentage of aberrant nuclei from three independent experiments per group was plotted and expressed as the mean ± SD (n = 100 cells per condition). Statistical differences were calculated using the unpaired t-test test; * p < 0.05.
